# Hybrid Aminoferrocenes With Nitrate Esters for In Situ Peroxynitrite Generation: Complex Interplay Between Redox Chemistry, NO‐Donor Reactivity, and Aminoferrocene Activation Influences In Vitro Anticancer Activity

**DOI:** 10.1002/cbic.70448

**Published:** 2026-07-08

**Authors:** Hülya Gizem Özkan, Roman Selin, Paula Holst, Rainer Tietze, Christoph Alexiou, Andriy Mokhir

**Affiliations:** ^1^ Department of Chemistry and Pharmacy Chair Organic Chemistry II Friedrich‐Alexander University (FAU) of Erlangen‐Nürnber Erlangen Germany; ^2^ Department of Otorhinolaryngology, Head and Neck Surgery Else Kröner‐Fresenius‐Foundation‐Professorship Section of Experimental Oncology and Nanomedicine (SEON) University Hospital Friedrich‐Alexander‐University of Erlangen‐Nürnberg (FAU) Erlangen Germany

**Keywords:** aminoferrocene, cancer, nitrate ester, prodrug, reactive oxygen species

## Abstract

Aminoferrocene‐based prodrugs represent a promising class of redox‐active anticancer agents that can be selectively activated in oxidative cellular environments. However, activation of these systems typically relies on reactive oxygen species (ROS) and is therefore inefficient in intracellular compartments with low ROS levels, such as the cytoplasm. In this study, we explored strategies to enhance activation of organelle‐non‐biased aminoferrocene prodrugs through peroxynitrite (ONOO^−^). Using a previously reported aminoferrocene–coumarin fluorogenic probe (**11a**) and a set of aminoferrocene‐based prodrugs **13** and **20a** and ROS‐insensitive controls **20b** and **20c** earlier developed in our group, we demonstrated that ONOO^‐^ generated from the donor 3‐morpholinosydnonimine hydrochloride (SIN‐1) efficiently triggers cleavage of arylboronic acid groups and formation of the active aminoferrocenium species both in cell‐free systems and in cancer cells. Consistent with this mechanism, the anticancer activity of these previously described aminoferrocene‐based prodrugs was significantly enhanced in the presence of SIN‐1 across multiple cancer cell lines. To exploit this effect in a self‐contained system, we designed and synthesized a hybrid aminoferrocene prodrug bearing an organic nitrate ester moiety capable of releasing nitric oxide, which, upon reaction with endogenous superoxide anion radical, should form ONOO^−^. The resulting compound **7** and several control derivatives were prepared and characterized. Although the design was intended to promote intracellular ONOO^−^ formation and ROS‐mediated activation, biological studies revealed that the nitrate ester functionality itself is the dominant determinant of cytotoxicity. Mechanistic investigations showed that prodrug **7** is efficiently internalized by cancer cells and induces moderate mitochondrial ROS formation but strongly increases intracellular aldehyde levels, likely via intramolecular nitrate‐mediated oxidation at the benzylic position. These findings highlight the complex interplay between redox chemistry, NO‐donor reactivity, and aminoferrocene activation pathways. Overall, this work demonstrates that peroxynitrite can efficiently activate aminoferrocene prodrugs in cytoplasmic environments and provides new insights into the design of redox‐active organometallic therapeutics.

## 1 Introduction

A key property of ferrocene and its derivatives is the one‐electron oxidation to the ferrocenium cation. This allows a range of well‐established applications. For example, the Fc/Fc^+^ redox couple is a universal electrochemical reference standard for calibrating redox potentials and studying electron‐transfer kinetics [1, 3]. Ferrocene derivatives function as homogeneous redox mediators in electrosynthesis, shuttling electrons efficiently between electrodes and substrates, and as an electron‐transfer relay in chemical and enzymatic sensors, converting molecular recognition events into measurable electrochemical signals [4, 6]. Incorporation of ferrocenyl units into ligands and catalysts allows redox‐switchable catalysis [7]. The large electronic change accompanying Fc/Fc^+^ interconversion enables molecular switching, charge‐transport modulation, and memory behavior in molecular electronics, as well as redox‐controlled host–guest binding in supramolecular systems [8, 10]. Collectively, these applications stem from the unique combination of reversible single‐electron oxidation, kinetic stability of both oxidation states, fast self‐exchange rates, and synthetic tunability of ferrocene derivatives, making ferrocene one of the most important molecular redox platforms in chemistry [1, 2].

Ferrocenes are not yet broadly used in medicine. However, their potential is increasingly recognized, with several experimental drugs and prodrugs already reported and some advancing into early clinical evaluation. Ferrocene derivatives have been extensively investigated as anticancer, antimalarial, antimicrobial, and antiviral agents [11, 14]. For example, ferroquine, an organometallic chloroquine analog, has demonstrated antimalarial efficacy and favorable safety profiles in phase I clinical trials, particularly against drug‐resistant Plasmodium falciparum [15, 16]. Ferrocene‐tamoxifen hybrids (“ferrocifens”) have shown promising antiproliferative activity and are under pre‐clinical investigation as potential anticancer agents [11, 12]. Aminoferrocene‐based prodrugs designed to be activated in the oxidative environment of cancer cells have been developed and explored as ROS‐activated therapeutics [17, 20] and ferroptosis inducers [21, 22], offering selective cytotoxicity in high‐ROS cancer cells. Furthermore, ferrocenyl motifs have been incorporated into kinase inhibitors and other hybrid scaffolds to modulate biochemical targets implicated in disease [13, 23]. Ferrocene derivatives are generally well tolerated in vivo, exhibiting low intrinsic toxicity relative to many conventional chemotherapeutics [11, 13].

**FIGURE 1:**
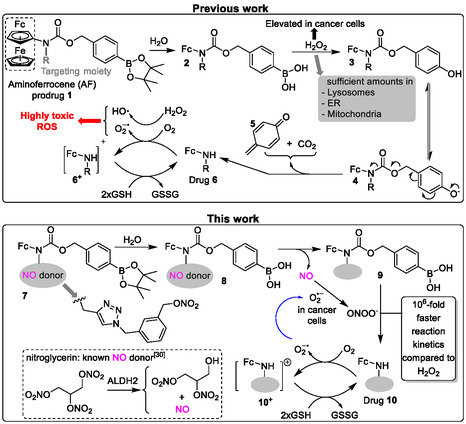
Upper panel (“Previous work”): Established mechanism of H_2_O_2_‐mediated activation of anticancer aminoferrocene (AF)‐based prodrugs with formation of highly toxic reactive oxygen species (ROS) occurring most efficiently in high‐ROS sites in cells: lysosomes, ER or mitochondria of cancer cells [24, 29]. Lower panel (“This work”): A concept of prodrugs (conjugates of AF‐prodrugs with organic nitric oxide donors [30]), which are activated in the cytoplasm of cancer cells, a relatively low‐ROS site in cells. The concept is based on in situ conversion of less reaction ROS (O_2_^•−^, H_2_O_2_) to more reactive one ONOO^−^.

The therapeutic potential of ferrocenes is partly attributed to their ability to undergo redox cycling between the Fc and Fc^+^ states, which can promote the generation of reactive oxygen species (ROS) and contribute to cytotoxic mechanisms [11, 13]. However, unspecific ROS induction can damage healthy tissues and provoke inflammatory responses, underscoring the importance of targeting or conditional activation. Our group has implemented ROS‐responsive strategies to address this challenge, including prodrugs that are activated by elevated hydrogen peroxide levels characteristic of pathological cells (Figure 1, “previous work”). In such systems, reaction with H_2_O_2_ leads to release of the active aminoferrocene (AF) drug that can catalytically generate highly toxic superoxide and hydroxyl radicals, enhancing selective destruction of disease cells while sparing normal tissues [17, 20]. Subsequent studies have shown that efficient activation of aminoferrocene prodrugs occurs preferentially in ROS‐rich organelles such as lysosomes [24, 25], endoplasmic reticulum [26], and mitochondria [27, 29], whereas activation in the relatively ROS‐poor cytoplasm and nucleus remains inefficient due to lower endogenous H_2_O_2_ concentrations. This limitation warrants research to improve prodrug activation in intracellular sites with lower oxidative stress.

In the present study, we partially addressed this limitation by exploring two interconnected strategies. In the first approach, we combined organelle‐non‐biased aminoferrocene prodrugs with the peroxynitrite (ONOO^−^) donor SIN‐1 and observed a substantial enhancement of anticancer efficacy. These findings motivated the development of a second strategy, in which we designed a hybrid aminoferrocene prodrug incorporating an organic nitrate ester moiety (Figure 1, “this work”). In this design, we exploited the well‐established ability of organic nitrate esters to function as nitric oxide (NO) donors, as exemplified by clinically used agents such as nitroglycerin [30]. Upon cellular internalization, NO released from the prodrug is expected to react with intracellular superoxide anions in a diffusion‐controlled process to generate peroxynitrite (ONOO^−^), a highly reactive oxidant. Notably, ONOO^−^ reacts with arylboronic acid trigger groups with kinetics approximately six orders of magnitude faster than those observed for H_2_O_2_ [31]. This property enables efficient prodrug activation and subsequent formation of a ROS‐generating aminoferrocene species even within ROS‐poor cytoplasmic compartments [32]. Collectively, these strategies expand the scope of ROS‐activated aminoferrocene prodrugs toward targeting cytoplasmic ROS‐sensitive biomolecules, which are typically inaccessible to conventional H_2_O_2_‐activated systems. Furthermore, this approach provides a potential platform for the selective modulation of oxidative stress in pathological cells.

## 2 Results and Discussion

### 2.1 Modulation of Activation and Anticancer Efficacy of Organelle‐Non‐Biased Aminoferrocene Prodrugs by Peroxynitrite

**FIGURE 2:**
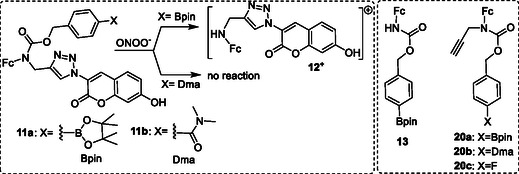
Structures of previously reported reference compounds **11a** [20], **11b** [33], **13** [17, 18], **20a–c** [20, 33]. The reaction of **11a** with ONOO^−^ is outlined in the left panel.

We selected the fluorogenic aminoferrocene‐7‐hydroxycoumarin conjugate **11a** and two aminoferrocene‐based prodrugs **13** and **20a** (Figure 2).

**FIGURE 3:**
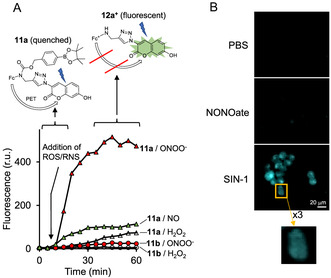
(A) Effect of ROS/RNS on fluorescence of compounds **11a** and **11b** (structures are shown in Figure 2): *λ*_ex_ = 370 nm, *λ*_em_ = 472 nm; [**11a**] = [**11b**] = 1 μM in PBS buffer. The compounds were first incubated for 10 min, followed by addition of ROS/RNS: NONOate (NO donor) or SIN‐1 (ONOO^−^ donor) or H_2_O_2_, each 0.5 mM. (B) Fluorescence microscopic images of A2780 cells pre‐incubated with 11a for 2 h, washed and incubated with either NONOate or SIN‐1 (both 0.5 mM) or PBS (negative control): *λ*_ex_ = 335–383 nm, *λ*_em_ = 420–470 nm.

According to our previous studies, these compounds do not exhibit preferential localization in specific organelles but are predominantly distributed in the cytoplasm [17, 20, 24]. First, we investigated the reaction of **11a** with SIN‐1, a donor of peroxynitrite (ONOO^−^), using fluorescence spectroscopy. Compound **11a** is non‐fluorescent in aqueous buffers because photoinduced electron transfer (PET) from the ferrocenyl moiety to the excited state of 7‐hydroxycoumarin efficiently quenches the dye fluorescence. Upon cleavage of the B—C bond, followed by 1,6‐elimination and CO_2_ release, an aminoferrocene derivative is formed (Figure 1, “previous work”). This species is highly electron‐rich and is readily oxidized by oxygen or other oxidants present in the reaction mixture to generate the corresponding aminoferrocenium derivative **12** ^+^ (Figure 2). The latter species cannot support PET, resulting in recovery of the fluorescence of 7‐hydroxycoumarin. This mechanism was previously demonstrated for the reaction of **11a** with H_2_O_2_ (Figure 3A) [34].

Notably, diethylamine diazeniumdiolate (NONOate), a nitric oxide donor, also induces an increase in fluorescence of **11a** (Figure 3A). However, in this case the mechanism involves direct NO‐mediated decomposition of the ferrocenyl moiety, as described previously [33]. Importantly, formation of the aminoferrocenium species (as observed in the presence of H_2_O_2_) is essential for the anticancer activity of aminoferrocene‐based prodrugs [17, 20], whereas NO‐induced ferrocenyl decomposition leads to prodrug deactivation [33].

Exposure of **11a** to ONOO^−^ generated from SIN‐1 resulted in a pronounced fluorescence increase (Figure 3A). Analysis of the reaction mixture by electrospray ionization quadrupole mass spectrometry (ESI‐Q MS) confirmed the formation of the aminoferrocenium intermediate **12** ^+^ (found *m/z* = 442; calcd. for C_22_H_18_FeN_4_O_3_ [M–e^−^]^+^, 442). This observation indicates that the reaction cascade is initiated by ONOO^−^‐mediated B—C bond cleavage, analogous to the H_2_O_2_‐triggered process shown in Figure 1 (“previous work”), rather than by undesired direct decomposition of the ferrocene unit as observed with NO donors. This interpretation is further supported by experiments with the ROS‐insensitive control compound **11b**, in which the boronic acid pinacol ester group is replaced by an *N,* *N*‐dimethylamide fragment [33]. Compound **11b** shows negligible response to ONOO^−^ (Figure 3A). Importantly, under identical conditions (0.5 mM SIN‐1, NONOate, or H_2_O_2_), SIN‐1 produced the strongest fluorescence response in the presence of **11a**, reaching a maximal fluorescence intensity of *F*_max_ = 500 ± 17 relative units (r.u.), compared with *F*_max_ = 99 ± 2 r.u. for NONOate and *F*_max_ = 72 ± 1 r.u. for H_2_O_2_ (Figure 3A).

Next, we examined whether ONOO^−^‐mediated activation of **11a** also occurs in cancer cells. Human ovarian cancer A2780 cells were selected because it is known that **11a** is efficiently accumulated in these cells and weakly activated in the presence of endogenous ROS [17, 20]. Cells were incubated with **11a** for 2 h, washed, and subsequently treated with SIN‐1, NONOate, or phosphate‐buffered saline (PBS) as a negative control. Under these conditions, only cells treated with SIN‐1 displayed fluorescence at excitation/emission wavelengths corresponding to activated **11a** (*λ*_ex_ = 335–383 nm, *λ*_em_ = 420–470 nm; Figure 3B). The fluorescence signal was distributed throughout the cytoplasm rather than localized to specific organelles (see the zoomed‐in image in Figure 3B), confirming that activation occurs in the relatively ROS‐poor cytoplasmic environment.

**FIGURE 4:**
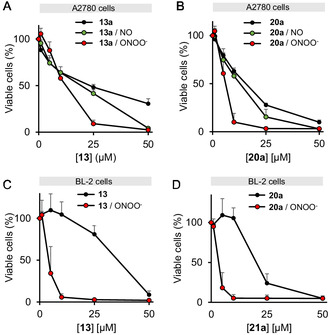
Effects of compounds **13** and **20a** on viability of ovarian cancer (A2780, insets A,B) and Burkitt's lymphoma (BL‐2, insets C,D) and cells in the absence and presence of non‐toxic concentrations of an NO donor (NONOate) or an ONOO^−^ donor (SIN‐1). Additional data are provided in Table 1. In all cases, at least three independent experiments were conducted (*N* = 3). Mean values are shown; error bars represent standard deviations (SD).

We next evaluated whether ONOO^−^ enhances the anticancer activity of the organelle‐non‐biased prodrugs **13** and **20a** in several ROS‐rich cancer cell lines, including A2780 (CVCL_0134), Burkitt's lymphoma BL‐2 (CVCL_1966), and human prostate cancer DU‐145 cells (CVCL_0105) (Figure 4 and Table 1). Three cancer cell lines were used to confirm that the observed effects are general. As a control, the ROS‐insensitive compound **20c** was also tested (Figure 2). The effects of SIN‐1 were compared with those of NONOate using non‐toxic concentrations of both reagents (0.5 mM; entries 1 and 2 in Table 1). The ONOO^−^ donor significantly potentiated the anticancer activity of both prodrugs (**13** and **20a**) in most cases, with one exception: the effect of **13** in DU‐145 cells, which are known to be particularly resistant to chemotherapy. For example, in the most sensitive BL‐2 cells, SIN‐1 increased the anticancer activity of weakly active prodrugs **13** (IC_50_ = 34 ± 4 μM) and **20a** (IC_50_ = 20.7 ± 0.5 μM) up to the level of the most successful lysosome‐targeting aminoferrocene‐based prodrug NCure2 [24] −4.2 ± 0.4 μM (entries 4 and 7, Table 1). In contrast, NONOate produced only minor effects. Interestingly, the activity of the lysosome‐targeting NCure2 [24] was largely unaffected by SIN‐1, except for a slight potentiation in DU‐145 cells (Table 1). These results suggest that endogenous H_2_O_2_ levels in lysosomes may already be sufficient for full activation of this lysosome‐localized prodrug.

**TABLE 1:** Anticancer efficacy of AF‐based prodrugs and their mixtures with NO and ONOO^−^ donors in vitro.

Compounds	IC_50_, μM/cell lines		
	A2780	BL‐2	DU‐145
1. DEA NONOate (NO donor)	>1000	>500	>1000
2. Sin‐1 Chloride (ONOO donor)	>1000	217 ± 87	>1000
3. **13**	24 ± 3	34 ± 4	>50
4. **13 **+ Sin‐1 Chloride	12 ± 2	3.5 ± 0.4	>50
5. **20a**	14 ± 1	20.7 ± 0.5	>50
6. **20a **+ DEA NONOate	12 ± 2	—	—
7. **20a **+ Sin‐1 Chloride	6 ± 2	3.4 ± 0.7	16 ± 3
8. **20c**	17 ± 2	19 ± 2	>50
9. **20c **+ Sin‐1 Chloride	17 ± 3	19 ± 3	>50
10. NCure2	7 ± 1	4.2 ± 0.4	20 ± 3
11. NCure2 + Sin‐1 Chloride	6.5 ± 0.4	3.0 ± 0.3	13 ± 4
12. **7**	6 ± 2	8 ± 2	—
13. **21a**	18 ± 2	18.5 ± 0,6	—
14. **21b**	5.0 ± 0.7	9 ± 2	—
15 **21c**	36 ± 1	38 ± 4	—

Collectively, these findings demonstrate that ONOO^−^ can efficiently activate aminoferrocene‐based prodrugs in the cytoplasm, a cellular compartment characterized by relatively low ROS levels.

### 2.2 Design and Synthesis of Prodrug 7 and Controls

Encouraged by the above‐described results confirming that ONOO^−^ efficiently activates aminoferrocene‐based prodrugs both in cell‐free settings and in cells, with formation of active ROS‐generating aminoferrocenium drugs, we attempted to make use of this effect to increase the anticancer efficacy of organelle‐non‐biased prodrugs. In particular, we aimed at a conjugate of a prodrug with an NO donor. The idea was that in cells the NO donor will release NO, which will combine with endogenous O_2_^•−^ in a diffusion‐limited reaction leading to formation of ONOO^−^ in proximity to the prodrug. The latter reagent is supposed to facilitate the activation of the prodrug, thereby enhancing its anticancer effect. We selected organic nitrate esters as NO‐donors based on the established knowledge that they release NO intracellularly in the presence of aldol dehydrogenase 2 (ALDH2) enzyme [35].

**FIGURE 5:**
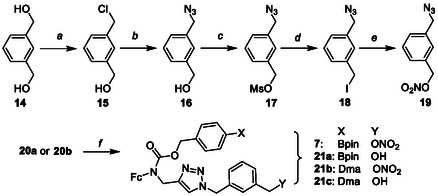
Synthesis of AF prodrug with an NO‐donor group and control compounds: (a) HCl (conc), toluene; (b) NaN_3_, DMSO; (c) Ms_2_O, Et_2_N, DCM; (d) LiI, DMSO; (e) AgNO_3_, ACN; (f) CuI, TBTA, DMF.

The desired conjugate (prodrug **7**) was prepared as outlined in Figure 5. As a first step the NO‐donor fragment 3‐(azidomethyl)benzyl nitrate (**19**) was prepared starting from 1,3‐benzenedimethanol (**14**), which was initially converted to the corresponding chloromethyl derivative **15** by treatment with hydrochloric acid in toluene, followed by nucleophilic substitution of the chloride for azide, subsequent mesylation of the resulting alcohol **16** with methanesulfonyl chloride (**17**), displacement of the mesylate with iodide furnishing the corresponding iodide **18**, which was finally treated with silver nitrate to obtain desired intermediate. In the last step aminoferrocene‐based prodrug **20a** was coupled with **19** under the conditions of a copper(I)‐catalyzed azide–alkyne “click” reaction to afford prodrug **7**. Control compounds **21a**–**c**, also newly synthesized, were prepared by analogous procedures as detailed in the Experimental Section. All new compounds were >95% pure according to elemental (C, H, N) analysis conducted using Vario Micro Cube (Elementar) instrument.

**FIGURE 6:**
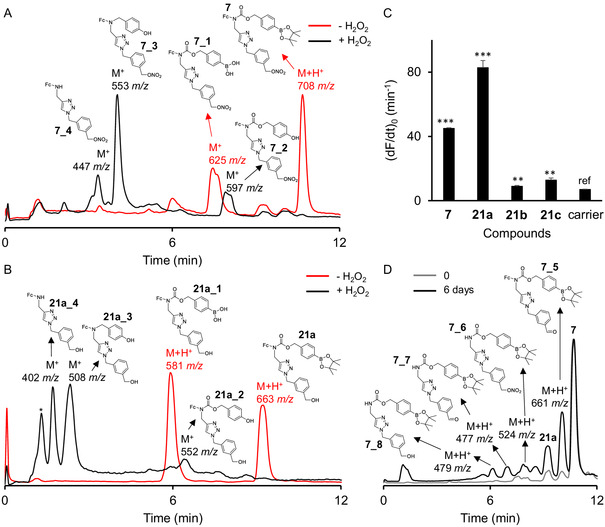
(A,B) HPLC‐MS analysis of mixtures of either prodrug **7** (30 μM, inset A) of control **21a** (30 μM, inset B) in the absence (red traces) or presence of H_2_O_2_ (10 mM, black traces) in triethylammonium acetate (TEAA) buffer (pH 7.4) containing ACN (23%, v/v) and DMSO (1%, v/v) after 10 min incubation at 37 °C. (C) Initial increase of fluorescence ((dF/dt)_0_, *λ*_ex_ = 501 nm, *λ*_em_ = 525 nm) of aqueous 2′,7′‐dichlorofluorescin (DCFH) solution containing 3‐(*N*‐morpholino)propanesulfonic acid buffer (MOPS buffer, 100 mM, pH 7.4), *N,* *N,N’,* *N*’‐ethylenediaminetetraacetic acid (EDTA, 10 mM), glutathione (GSH, 5 mM), and H_2_O_2_ (10 mM) in the absence or presence of different ferrocene derivatives as indicated on the plot. Carrier: 1% DMF in water (v/v). The value (dF/dt)_0_ correlates with the rate of ROS generation. (D) HPLC‐MS analysis of stability of DMSO solution of prodrug **7** (3 mM) after 6 day—incubation at 22 °C (black trace). The freshly prepared probe analysis (gray trace) is given for comparison. Unpaired Student's *t* test was used to compare mean values with that obtained for the “carrier” sample indicated as “ref”: **—*p *< 0.01; ***—*p *< 0.001.

The solubility of prodrug **7** in aqueous phosphate‐buffered saline (PBS, pH 7.4) is 10 μM, which is consistent with its relatively high lipophilicity (log*P *= 5.39 ± 0.26). In aqueous solution, the prodrug undergoes rapid hydrolysis to form the corresponding boronic acid derivative **7_1** (Figure 6A), which is less lipophilic (log*P *= 3.37 ± 0.24).

Consistent with the relatively high lipophilicity of both **7** and its hydrolysis product **7_1**, their apparent solubility increased markedly in Roswell Park Memorial Institute medium (RPMI 1640) supplemented with 5% fetal bovine serum (FBS), reaching 104 μM. Similar trends were observed for the control compounds **21a**–**c**. These two aqueous media were therefore used in the experiments described in this study.

### 2.3 Activation of Prodrug 7 in Cell‐Free Settings

We used HPLC coupled with ESI‐Q‐MS to evaluate the transformation of prodrug **7** and control compound **21a**, which lacks the nitrate ester moiety, in the absence and presence of H_2_O_2_ (Figure 6A,B). In both cases, we observed the expected formation of the aminoferrocene drugs **7_4** (from prodrug **7**) and **21a_4** (from control **21a**). The detected intermediates (**7_1**, **7_2**, and **7_3**) are consistent with the activation pathway previously reported for other aminoferrocene‐based prodrugs (Figure 1). These results indicate that the reaction of H_2_O_2_ with the arylboronic acid moiety of **7** proceeds independently of the presence of the nitrate ester fragment.

Next, we investigated whether prodrug **7** can promote ROS generation under cell‐free conditions by monitoring the oxidation of 2′, 7′‐dichlorofluorescin in the presence of H_2_O_2_. Both boronic acid derivatives (**7** and **21a**) proved to be efficient catalysts for this reaction, accelerating the oxidation 6.4‐fold and 11.7‐fold, respectively, relative to the reaction in the absence of these compounds (Figure 6C). In contrast, the ROS‐insensitive controls **21b** and **21c**, which lack the H_2_O_2_‐responsive boronic acid group and therefore cannot form catalytically active aminoferrocene species, increased the reaction rate by less than 1.5‐fold.

In a separate experiment, we examined whether prodrug **7** can spontaneously generate nitric oxide (NO). For this purpose, DMSO solutions of **7** were stored at 22 °C for 6 days and analyzed by HPLC–MS. As a control, an identical solution was stored at −20 °C, under which conditions the prodrug remained stable (data not shown). In contrast, storage at 22 °C resulted in partial decomposition of **7** (Figure 6D). The degradation primarily involved the nitrate ester moiety, yielding its hydrolysis product (**21a**, the corresponding alcohol) and its oxidation product, the aldehyde **7_5**. Formation of the aldehyde can be rationalized by an intramolecular oxidation process in which the nitrate ester acts as an electron acceptor, which would concomitantly generate NO. Consistent with this hypothesis, we also detected prominent peaks corresponding to NO‐mediated cleavage of the ferrocene moiety [33], giving products **7_6**, **7_7**, and **7_8**.

The control compound **21b**, which lacks the ROS‐responsive boronic acid group, exhibited decomposition behavior similar to that of prodrug **7**, whereas the controls **21a** and **21d**, both lacking the nitrate ester functionality, remained stable. These observations indicate that the nitrate ester group can undergo slow, enzyme‐independent decomposition, resulting in spontaneous NO formation.

### 2.4 Anticancer Effect In Vitro and the Mechanism of Action

**FIGURE 7:**
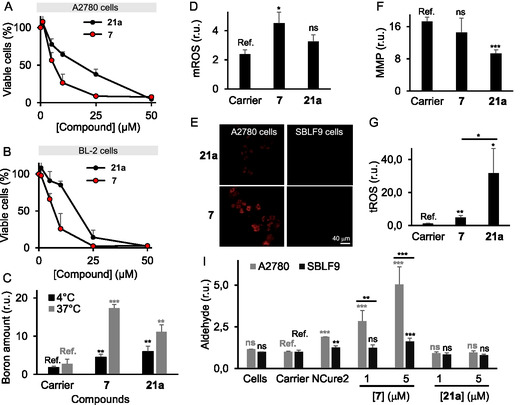
(A,B) Effect of prodrug **7** and control **21a** on viability of human ovarian cancer A2780 cells (inset A) and Burkitt's lymphoma BL‐2 cells (inset B). IC_50_ values for these and other compounds are given in Table 1. (C) Uptake of prodrug **7** and control **21a** by A2780 cells at 4 °C and 37 °C determined by analysis of intracellular boron amount by using atomic emission spectroscopy (AES). (D,E) Effects of prodrug **7** and control **21a** on the level of mitochondrial ROS (mROS) in A2780 cells determined by flow cytometry (inset D) and in A2780 and SBLF9 cells by using fluorescence microscopy (inset E). (F,G) Effects of prodrug **7** and control **21a** on mitochondrial membrane potential (MMP, inset F) and the level of total ROS (tROS, inset G) in A2780 cells. (I) Effect compounds and controls on the amount of intracellular aldehydes in A2780 cells. Carrier: aqueous DMSO solution (1%, v/v). An unpaired Student's *t*‐test was performed for statistical analysis of the data: *—*p *< 0.05; **—*p *< 0.01; ***—*p *< 0.001; ns—*p *≥ 0.05.

We investigated the effect of prodrug **7** and a series of control compounds (**21a–c**) on the viability of A2780 and BL‐2 cells (Figure 7A,B and Table 1, entries 12–15). Prodrug **7** was substantially more active than control **21a**, which lacks the nitrate ester moiety, in both cell lines (IC_50_ = 6–8 μM vs. 18–18.5 μM, respectively). However, another control compound, **21b**, in which the nitrate ester group was retained but the ROS‐sensitive boronic acid moiety was replaced with a ROS‐resistant *N*,*N*‐dimethylamide group, exhibited similar cytotoxic activity to that of prodrug **7**. Finally, compound **21c**, lacking both the nitrate ester and the ROS‐sensitive moiety, was the least toxic compound in this series (Table 1). These results indicate that the nitrate ester functionality is primarily responsible for the anticancer activity, whereas ROS‐mediated prodrug activation appears to play only a minor role.

Prodrug **7**, rather than compound **21b**, was selected for further investigation of its mechanism of action. This choice was based on the slightly higher solubility of compound **7** in aqueous buffers and its greater stability in the solid state. The biological effects of compound **7** were therefore compared with those of control **21a**, which lacks the nitrate ester moiety. To investigate the cellular uptake mechanism of prodrug **7**, we quantified the intracellular boron content in A2780 cells following treatment with the compound (Figure 7C). Because the endogenous boron level in mammalian cells is close to zero, the measured boron concentration directly reflects the efficiency of prodrug uptake. Treatment of A2780 cells with prodrug **7** at 37 °C resulted in a strong increase in intracellular boron levels (Student's *t*‐test, *p *< 0.001). When the experiment was performed at 4 °C, the intracellular boron level remained higher than in untreated cells (*p *< 0.01), but was reduced by approximately 3.8‐fold compared with uptake at 37 °C (*p *< 0.001). These findings indicate that prodrug **7** is predominantly internalized via an energy‐dependent (active) uptake mechanism, which is strongly inhibited at low temperature, although a minor but significant contribution from a temperature‐independent passive process is also present. In contrast, control compound **21a** is taken up mainly through passive diffusion, with only a minor contribution from an active component, as indicated by the relatively low temperature dependence of its uptake: lowering the temperature from 37 °C to 4 °C decreased uptake efficiency by only 1.9‐fold (*p *< 0.05).

Prodrug **7** increased the level of mitochondrial reactive oxygen species (mROS) in A2780 cells by approximately 1.8‐fold (*p *< 0.05; Figure 7D,E). This moderate effect was not observed for control compound **21a**. Importantly, prodrug **7** did not affect mROS levels in healthy SBLF9 cells, indicating cancer‐cell selectivity (Figure 7E). Unlike control compound **21a**, prodrug **7** did not significantly alter the mitochondrial membrane potential (MMP) (Figure 7F). Aminoferrocene‐based drugs typically perturb MMP, thereby triggering increased mROS production [25, 34]. In the present case, however, this mechanism does not appear to operate. The biological effects of compound **7** do not seem to be confined to mitochondria. Treatment of A2780 cells with **7** also increased total intracellular ROS (tROS) levels (Figure 7G). However, this effect was more pronounced for compound **21a**.

Prodrug **7** may generate aldehydes in cells via two potential mechanisms. First, it may induce intracellular ROS formation, which can react with proteins and lipids to produce corresponding aldehydes. Second, aldehydes may be generated directly via intramolecular oxidation of the benzylic position by the nitrate ester moiety. The latter pathway was demonstrated for prodrug **7** in cell‐free experiments as described above (Figure 6D). Consistent with this mechanism, treatment of A2780 cells with compound **7** produced a dramatic increase in intracellular aldehyde levels, detectable already at 1 μM (*p* < 0.001) and further enhanced at 5 μM (*p *< 0.001) (Figure 7I). Notably, the reference compound NCure2, which is a substantially stronger intracellular ROS inducer [24], generated significantly lower levels of intracellular aldehydes even at a ten‐fold higher concentration (*p *< 0.05). These results strongly suggest that direct aldehyde formation via intramolecular oxidation represents the dominant pathway for compound **7**. This interpretation is further supported by the observation that control compound **21a**, which lacks the nitrate ester and generates considerably higher levels of tROS than **7** (Figure 7G), does not induce an increase in intracellular aldehydes (Figure 7I).

Despite the apparent independence of its cytotoxicity from ROS generation (cf. the similar toxicities of compounds **7** and **21b**, Table 1), prodrug **7** displays clear cancer‐cell selectivity. In addition to increasing mROS levels, compound **7** also elevates the level of intracellular aldehydes in cancer A2780 cells but not in healthy SBLF9 cells (Figure 7I).

A broad range of ferrocene‐containing compounds has been reported to exhibit antiproliferative activity, often in the low‐micromolar concentration range, and in some cases even reaching nanomolar potency depending on structural context and target engagement. Notably, ferrocene‐based systems such as ferrocifens, ferrocene‐tamoxifen hybrids display diverse and concentration‐dependent mechanisms of action involving redox activation and formation of reactive intermediates [11], while other ferrocenyl derivatives and hybrids have been shown to exert cytotoxic effects through a variety of pathways, including enzyme inhibition and intracellular redox imbalance [36]. In several cases, significant antiproliferative activity has been attributed, at least in part, to the intrinsic redox properties of the ferrocenyl moiety itself, which can undergo reversible oxidation to the corresponding ferrocenium species and participate in biologically relevant electron‐transfer processes [37]. These observations highlight that potent activity can be achieved without the need for explicit prodrug activation strategies. In contrast, the aminoferrocene‐based systems investigated in this work are designed to function as redox‐responsive prodrugs that require oxidative conversion to the corresponding aminoferrocenium species for activity. While this approach may inherently limit potency to the low‐micromolar range, it offers the potential advantage of controlled activation under specific intracellular conditions. Within this context, the activity of compounds **7** and **21a**–**c** is comparable to that of many reported ferrocenyl anticancer agents [11, 36, 37], while providing additional mechanistic control through stimulus‐dependent activation, which may be advantageous for achieving selectivity in complex biological environments.

## 3 Conclusion

In this study, we investigated strategies to enhance the activation and anticancer efficacy of aminoferrocene‐based prodrugs in cellular environments with relatively low levels of endogenous reactive oxygen species. Using a fluorogenic aminoferrocene probe, we demonstrated that peroxynitrite efficiently cleaves arylboronic acid trigger groups and generates the corresponding aminoferrocenium species both in cell‐free systems and in cancer cells. Consistent with this observation, the peroxynitrite donor SIN‐1 strongly potentiated the anticancer activity of several organelle‐non‐biased aminoferrocene prodrugs in multiple cancer cell lines, confirming that ONOO^−^ can serve as an efficient activator of these systems in the cytoplasm.

To translate this concept into a single‐molecule design, we synthesized a hybrid prodrug **7** combining an aminoferrocene ROS‐generating scaffold with an organic nitrate ester moiety capable of releasing nitric oxide. The initial hypothesis was that intracellular NO release would react with endogenous superoxide to generate ONOO^−^ in situ, thereby promoting activation of the boronic acid trigger group. However, biological evaluation of prodrug **7** and control derivatives revealed that the nitrate ester functionality itself largely determines cytotoxicity, whereas ROS‐mediated activation of the aminoferrocene moiety contributes only marginally under the studied conditions. Mechanistic investigations further showed that prodrug **7** is taken up efficiently by cancer cells through a predominantly energy‐dependent pathway and selectively increases mitochondrial ROS levels in cancer cells but not in healthy fibroblasts. Notably, treatment with compound **7** resulted in a pronounced intracellular accumulation of aldehydes, which appears to arise mainly from intramolecular nitrate‐mediated oxidation rather than from ROS‐induced lipid or protein oxidation. These findings reveal an additional, previously not reported reactivity pathway for nitrate ester‐containing aminoferrocene derivatives.

Overall, this work demonstrates that peroxynitrite is a powerful activator of aminoferrocene‐based prodrugs and highlights the potential of combining redox‐active organometallic scaffolds with reactive nitrogen oxide donors. At the same time, the results underline the importance of carefully considering competing chemical pathways when designing multifunctional redox‐active therapeutics. The insights obtained here will facilitate the future development of ferrocenyl prodrugs capable of selective activation in diverse intracellular environments.

## 4 Experimental Section

### 4.1 General

Commercially available chemicals of the best quality from Sigma–Aldrich (Germany) and Alfa–Aesar (Germany) were obtained and used without purification. NMR spectra were acquired on a Bruker Avance 300 (Ettlingen, Germany) or Bruker Avance 400 (Ettlingen, Germany). ESI mass spectra were recorded on a Bruker ESI MicroTOF II (Bremen, Germany) or Bruker maXis 4G mass spectrometers (Bremen, Germany). C/H/N elemental analysis was performed in the microanalytical laboratory of the Department Chemistry and Pharmacy, Organic Chemistry Chairs 1 and 2 of the Friedrich‐Alexander‐University of Erlangen‐Nuremberg. UV–visible spectra were measured on a Cary 100 UV–visible spectrophotometer (Agilent Technologies, Frankfurt am Main, Germany) by using either quartz glass cuvettes (Hellma GmbH, Müllheim, Germany) with a sample volume of 1 mL or micro‐cuvettes with a sample volume of 100 μL (BRAND GmbH, Wertheim, Germany). The fluorescence images of live cells were acquired by using a Zeiss Axio Vert.A1 microscope (Jena, Germany) using 40×/1.30 objective (oil DIC). Statistical analysis of the data was conducted by using Student's *t‐*test. Two data sets were considered significantly different from each other for *p *< 0.05.

### 4.2 Preliminary Experiments

Reference compounds **11a** [24], **11b** [33], **13** [17, 18], **20a** [20], **20b** [33], **20c** [20], and *N*‐ferrocenyl‐N‐[4‐(1‐piperidinylmethyl)benzyl]aminocarboyl‐oxymethylphenylboronic acid pinacol ester (NCure2) [24] were prepared as described elsewhere.

Activation of fluorogenic, ROS‐responsive prodrug **11a**, and ROS‐insensitive control **11b** under different conditions in cell‐free settings

Compounds were dissolved in DMSO, and 10 µL of this stock solution was diluted in phosphate‐buffered saline (PBS: phosphate 10 mM, NaCl 150 mM) to obtain 1 µM solution containing DMSO (1%, v/v). After 10 min, either of the activators NONOate (NO donor) or SIN‐1 (ONOO^−^ donor) or H_2_O_2_ was added. The final concentration of each reagent was 500 μM. The reaction was monitored by fluorescence spectroscopy for 120 min: *λ*_ex_ = 370 nm, *λ*_em_ = 472 nm.

Activation of **11a** under different conditions in A2780 cells

A2780 cells were seeded on a 35 mm imaging dish (*µ*‐Dish 35 mm, high, ibidi GmbH, Germany) at a cell density of 80 cells/µL 1 day before the experiment in 2000 µL RPMI 1640 medium containing 5% FBS, 1% L‐glutamine, and 1% penicillin/streptomycin. On the next day, the cells were washed with DPBS (2 × 2 mL). A fresh portion of RPMI 1640 medium (2 mL) was added. Compound **11a** in DMSO (final concentration 25 µM) was added and incubated in the incubator at 37 °C containing 5% CO_2_ for 2 h. Afterward, the cells were washed, and either DEA‐NONOate (NO donor; 0.5 mM in PBS) or SIN‐1 chloride (ONOO donor; 0.5 mM in PBS) was added, and the cells were further incubated for 30 min. As a control, the cells were first incubated with pure DMSO only and then with PBS. Finally, the cells were washed with DPBS (2 × 2 mL), and the fresh medium (2 mL) was added. The fluorescence images were taken with a Zeiss Axio Vert.A1 and filter set: *λ*_ex_ = 335–383 nm, *λ*_em_ = 420–470 nm. Objective: 40×/1.30. Oil DIC.

Effect of SIN‐1 on anticancer efficacy of reference compounds **13**, **20a**, **20c**, and NCure2

Burkitt lymphoma (BL‐2) cells were obtained from DSMZ (Germany) and grown in RPMI 1640 medium supplemented with 20% fetal bovine serum (FBS), 1% L‐glutamine, and 1% penicillin/streptomycin. BL‐2 cells were grown to (0.5–1.5) × 10^6^ cells/mL and diluted as required. Human ovarian cell line (A2780) was obtained from Sigma–Aldrich and grown in RPMI 1640 medium supplemented with 10% FBS, 1% L‐glutamine, and 1% penicillin/streptomycin. A2780 cells were cultivated to 80%–90% confluence and detached from the flask by using trypsin/EDTA solution (0.025%/0.01%, w/v, Sigma Aldrich GmbH, Germany) in PBS.

A2780 and BL‐2 cells were resuspended in the RPMI 1640 medium containing 5% FBS. The cell suspensions (100 µL/well) were added to a 96‐well microtiter plate: 50,000 cells/100 µL for BL‐2 and 25,000 cells/100 µL for A2780 cells. A2780 cells were seeded 1 day before for attachment and kept at 37 °C in the chamber filled with CO_2_ (5%) overnight. Next, the stock solutions of the reference compounds dissolved in DMSO at 5, 2.5, 1, 0.5, and 0.1 mM were added to the wells, giving rise to final concentrations of 50, 25, 10, 5, and 1 µM, and preincubated for 150 min. Afterward, freshly dissolved PBS solutions of either SIN‐1 or NONate were added and incubated for further 48 h. Final concentrations of SIN‐1 were 50 µM for BL‐2 and 100 µM for A2780 cells, whereas the final concentration of NONOate was 100 µM for both cell lines. For determination of the anticancer efficacy of the reference compounds, they were incubated with cells without any additives for 48 h. Pure DMSO containing either SIN‐1 or NONOate were used as blanks. Afterward, 20 μL/well 3‐(4,5‐dimethylthiazol‐2‐yl)‐2,5‐diphenyltetrazolium bromide (MTT) solution (5 mg/mL in DPBS) was added. After another 3 h incubation under the same conditions, the cells were treated with 90 μL/well sodium dodecyl sulfate (SDS, 10% solution in 0.01 M aqueous HCl) and incubated overnight. The next day, the intensity of absorbance at 590 nm was measured and the absorbance at 690 nm was taken as a baseline value using the plate reader (Mithras LB940, Berthold, Bad Wildbad, Germany). These data were used to calculate the relative number of viable cells.

### 4.3 Synthesis

(3‐(Chloromethyl)phenyl)methanol (**15**)

1,3‐Phenylenedimethanol **14** (5.000 g, 36.19 mmol) in toluene (180 mL) and 32% hydrochloric acid (18.5 mL, 589 mmol) was stirred at 22 °C overnight. The layers were separated, and the organic layer was washed with water (2 × 150 mL) and dried over anhydrous magnesium sulfate. The solvent was removed under reduced pressure to obtain the product as colorless oil (3.182 g, 20.32 mmol, 56.1%), spectroscopy (300 MHz, DMSO‐d_6_) *δ* 7.42–7.37 (m, 1H), 7.34–7.23 (m, 3H), 5.23 (t, *J* = 5.7 Hz, 1H), 4.76 (s, 2H), 4.50 (dd, *J* = 5.7, 0.7 Hz, 2 H).

(3‐(Azidomethyl)phenyl)methanol (**16**)

Compound **15** (1.506 g, 9.61 mmol) was dissolved in DMSO (25 mL). Sodium azide (690 mg, 10.61 mmol) was added to the solution and the reaction mixture was stirred at 22 °C overnight. After the reaction appeared to be completed (according to ^1^H NMR spectroscopy), water (30 mL) was added, and the solution was extracted with diethyl ether (3 × 150 mL). The combined organic layers were washed with water (3 × 150 mL) and dried over anhydrous magnesium sulfate. The solvent was removed under reduced pressure to obtain the product as colorless oil (1.447 g, 8.87 mmol, 92.3%). ^1^H NMR spectroscopy (400 MHz, DMSO‐d_6_) *δ* 7.39–7.26 (m, 3H), 7.24–7.20 (m, 1H), 5.23 (t, *J* = 5.7 Hz, 1H), 4.51 (dd, *J* = 5.7, 0.7 Hz, 2H), 4.44 (s, 2H).

3‐(Azidomethyl)benzyl methanesulfonate (**17**)

The reaction was performed under N_2_ atmosphere. Compound **16** (149.0 mg, 913.1 μmol) was dissolved in anhydrous dichloromethane (DCM, 10 mL). At a temperature of 0 °C, triethylamine (134 μL, 967 μmol) was added. Methanesulfonic anhydride (166.9 mg, 958.1 μmol) was dissolved in anhydrous DCM (3 mL), and added dropwise to the reaction mixture. The reaction mixture was stirred at 0 °C for 10 min and allowed to warm up to 22 °C afterward. After 40 min, the reaction appeared to be completed according to ^1^H NMR spectroscopy. It was diluted with DCM (20 mL) and washed with water (3 × 30 mL). The organic layer was dried over anhydrous magnesium sulfate and the solvent was removed under reduced pressure. The product was obtained as colorless oil (205.2 mg, 850.5 μmol, 93.1%). ^1^H NMR spectroscopy (400 MHz, DMSO‐d_6_) *δ* 7.50–7.38 (m, 4H), 5.28 (s, 2H), 4.50 (s, 2H), 3.24 (s, 3H).

3‐(Azidomethyl)benzyl nitrate (**19**)

Compound **17** (205 mg, 850 μmol) was dissolved in DMSO (10 mL). LiI (342 mg, 2.55 mmol) was added. The reaction mixture was stirred at 22 °C for 6 h. After the reaction appeared to be completed according to ^1^H NMR spectroscopy, it was diluted with water (20 mL). The aqueous layer was extracted with diethyl ether (4 × 30 mL) and the combined organic layers were washed with water (3 × 50 mL). The solvent was evaporated under reduced pressure. After column chromatography on silica gel (hexane/EtOAc 50/1, v/v), 1‐(azidomethyl)‐3‐(iodomethyl)benzene (**18**) was obtained: *R*_f_ (n‐hexane/EtOAc 50:1) = 0.29. Its purity was <95%. This intermediate was used in this crude form in the next step.

The next reaction was performed under N_2_ atmosphere and the reaction flask was protected from light. Compound **18** (85 mg, 0.31 mmol) was dissolved in anhydrous acetonitrile (ACN, 15 mL) and AgNO_3_ (132 mg, 0.779 mmol), dissolved in anhydrous ACN (5 mL), was added dropwise over 45 min at 22 °C. The reaction was completed after 18 h according to ^1^H NMR spectroscopy. Next, Et_2_O (30 ml) was added and the organic layer—washed with H_2_O (3 × 50 mL). ACN (30 mL) was added to the organic layer, which was then dried over anhydrous magnesium sulfate. The solvent was removed under reduced pressure until 30 mL of the solution remained. The product was not obtained in the dried form, since it is potentially explosive. The resulting solution was used in the next reaction step. Small amount of the sample was purified by column chromatography to characterize this compound by ^1^H NMR spectroscopy (400 MHz, DMSO‐d_6_): *δ* 7.50–7.39 (m, 4H), 5.59 (s, 2H), 4.50 (s, 2H).

Intermediates **18** and **19** are potentially explosive. Therefore, these products should not be obtained in dry form.

Prodrug **7.** The reaction was performed under N_2_ atmosphere. Compound **20a** [20] (149 mg, 299 μmol) was dissolved in anhydrous DCM (3.0 mL), and diisopropylethylamine (DIPEA, 260 μL, 1.49 mmol) was added. A solution of compound **19** in ACN was added. CuI (8.0 mg, 42 μmol) and tris[(1‐benzyl‐1H‐1,2,3‐triazol‐4‐yl)methyl]amine (TBTA, 16 mg, 30 μmol) were dissolved in anhydrous ACN (3.0 mL) and added to the reaction mixture. The reaction mixture was stirred at 22 °C overnight. After the reaction appeared to be completed according to thin layer chromatography (TLC, eluent: hexane/EtOAc 2/1, v/v), the solvent was removed and the crude product was purified by column chromatography on silica (hexane/EtOAc, from 2/1 to 3/2, v/v). The product was obtained as orange solid (91 mg, 129 μmol, 45%). TLC (SiO_2_, eluent hexane:EtOAc, 3/2, v/v) *R*_f_ = 0.38. ^1^H NMR spectroscopy (400 MHz, DMSO‐d_6_) *δ* 8.02 (s, 1H), 7.64 (d, *J* = 7.6 Hz, 2H), 7.45–7.29 (m, 6H), 5.61 (s, 2H), 5.52 (s, 2H), 5.17 (s, 2H), 4.92 (s, 2H), 4.54 (s, 2H), 4.06 (s, 5H), 4.01 (t, *J* = 1.9 Hz, 2H), 1.29 (s, 12H). ^13^C{^1^H} NMR spectroscopy (151 MHz, DMSO‐d_6_) *δ* 144.48, 139.64, 136.59, 134.48, 132.89, 129.14, 128.94, 128.76, 128.58, 127.04, 123.43, 100.53, 83.66, 74.64, 68.68, 66.72, 64.14, 62.31, 52.42, 45.36, 24.63. High resolution electrospray ionization time‐of‐flight mass spectrometry (HR‐ESI‐TOF), positive mode: found 708.2214 *m/z*; calcd. for C_35_H_38_BFeN_5_O_7_ [M + H]^+^: 708.2208. Elemental (C, H, N) analysis (%): found C: 59.20, H: 5.61, N: 9.78; calcd. for C_35_H_38_BFeN_5_O_7_: C: 59.43, H: 5.41, N: 9.90.

Control **21a** was obtained using a method similar to that of prodrug **7**, except that (3‐(azidomethyl)phenyl)methanol **16** was used instead of 3‐(azidomethyl)benzyl nitrate **19**. The product was obtained as orange solid (220 mg, 332 μmol, 53.9%). TLC (SiO_2_, eluent: hexane:EtOAc, 2/3, v/v) *R*_f_ = 0.35. ^1^H NMR spectroscopy (400 MHz, DMSO‐d_6_) *δ* 7.98 (s, 1H), 7.66 (d, *J* = 7.5 Hz, 2H), 7.39–7.23 (m, 5H), 7.14 (d, *J* = 7.3 Hz, 1H), 5.56 (s, 2H), 5.24–5.13 (m, 3H), 4.92 (s, 2H), 4.55 (s, 2H), 4.46 (d, *J* = 5.6 Hz, 2H), 4.08 (s, 5H), 4.01 (t, *J* = 2.0 Hz, 2H), 1.29 (s, 12H). ^13^C{^1^H} NMR spectroscopy (101 MHz, DMSO‐d_6_) *δ* 153.71, 144.51, 143.23, 139.76, 135.88, 134.63, 128.55, 127.23, 126.27 (d, *J* = 8.8 Hz), 125.99, 123.48, 100.60, 83.79, 68.83, 66.87, 64.28, 62.68, 62.36, 52.94, 25.05, 24.77. HR‐ESI‐MS, positive mode: found 662.2346 *m/z*; calcd. for C_35_H_39_BFeN_4_O_5_ 662.2364. Elemental (C, H, N) analysis (%): found – C: 63,21, H: 5,98, N: 8,67; calcd for C_35_H_39_BFeN_4_O_5_ – C: 63,47, H: 5,94, N: 8.46.

Control **21b** was obtained using similar to prodrug **7**, except that compound **20b** [33] was used instead of compound **20a** [20]. TLC: *R*_f_ = 0.32 (DCM/MeOH, 20/1, v/v). ^1^H NMR spectroscopy (601 MHz, acetone‐d_6_) *δ* 7.77 (s, 1H), 7.50–7.36 (m, 8H), 5.64 (s, 2H), 5.54 (s, 2H), 5.23 (s, 2H), 5.00 (s, 2H), 4.67 (s, 2H), 4.06 (s, 5H), 3.98 (t, *J* = 2.0 Hz, 2H), 3.00 (d, *J* = 31.6 Hz, 6H). ^13^C{^1^H} NMR spectroscopy (151 MHz, acetone‐d_6_) *δ* 170.96, 154.21, 146.16, 138.83, 137.78, 137.76, 134.32, 130.22, 129.97, 129.80, 129.65, 128.67, 128.16, 123.96, 102.18, 75.55, 69.64, 67.50, 65.09, 63.42, 53.85, 46.33, 39.49, 35.14. HR‐ESI‐MS, positive mode: found 653.1726 *m/z*; calcd. for C_32_H_33_FeN_6_O_6_ [M + H]^+^: 653.1727. Elemental (C, H, N) analysis (%): found C: 58.46, H: 5.19, N: 12.68; calcd for C_32_H_33_FeN_6_O_6_: C: 58.91, H: 4.94, N: 12.88.

Control **21c** was obtained using a method similar to that of prodrug **7**, except that compound **20b** [33] was used instead of compound **20a** [20] and (3‐(azidomethyl)phenyl)methanol **16** was used instead of 3‐(azidomethyl)benzyl nitrate **19**. The product was obtained as orange solid (36 mg, 55 μmol, 7.2%). TLC: *R*_f_ = 0.22 (DCM/MeOH, 20/1, v/v). ^1^H NMR spectroscopy (400 MHz, acetone‐d_6_) *δ* 7.76 (s, 1H), 7.45–7.35 (m, 4H), 7.34–7.29 (m, 3H), 7.24–7.17 (m, 1H), 5.58 (s, 2H), 5.22 (s, 2H), 5.00 (s, 2H), 4.67 (s, 2H), 4.57 (d, *J* = 6.0 Hz, 2H), 4.32 (t, *J* = 5.8 Hz, 1H), 4.07 (s, 5H), 4.00–3.96 (m, 2H), 3.00 (d, *J* = 20.0 Hz, 6H). ^13^C{^1^H} NMR spectroscopy (101 MHz, acetone‐d_6_) *δ* 171.08, 146.06, 144.30, 138.86, 137.62, 136.85, 129.50, 128.59, 128.16, 127.29, 127.18, 126.93, 123.83, 69.65, 67.47, 65.08, 64.29, 64.16, 58.44, 54.26, 39.57, 35.19. HR‐ESI‐MS (positive mode): found: 608.1885 *m/z*; calcd. for C_33_H_33_FeN_5_O_4_ [M + H]^+^: 608.1876. Elemental (C, H, N) analysis (%): found: C: 60.73, H: 5.71, N: 11.44; calcd. for C_33_H_33_FeN_5_O_4_: C: 63.27, H: 5.48, N: 11.53.

### 4.4 Solubility of Compounds in Aqueous Solutions

Compounds were dissolved in DMSO at concentrations of 20, 10, 5, 2, 1.5, 1, and 0.2 mM. These solutions (10 μL) were transferred to quartz cuvettes containing either phosphate‐buffered saline (PBS) or Roswell Park Memorial Institute (RPMI) 1640 medium containing 5% fetal bovine serum (FBS) (990 μL) to obtain solutions (or suspensions) with final concentrations of compounds—200, 100, 50, 20, 15, 10, and 2 μM. The blank was also prepared, which contained DMSO only. The absorbance at 790 nm (where no peak of any compound is observed) of the resulting solutions/suspensions was measured and plotted as a function of concentrations of the compounds. The concentration at which the absorbance at 790 nm reaches twice that of the blank absorbance (0.04) was considered the solubility limit. The solubility was calculated based on at least 3 independent experiments (Table 2).

**TABLE 2:** Solubility of compounds in two different aqueous solutions.

Compound	Solubility	
	PBS, µM^a^	RPMI 5% FBS, µM^b^
**7**	10^c^	104 ± 12
**21a**	10^c^	94 ± 10
**21b**	19 ± 2	58 ± 6
**21c**	104 ± 9	160 ± 35

a PBS: phosphate‐buffered saline, pH 7.4.

b RPMI (5% FBS): Roswell Park Memorial Institute (RPMI) 1640 medium containing 5% of fetal bovine serum (FBS).

c Precipitation was observed at concentration of 30 µM.

### 4.5 n‐Octanol/Water Partition Coefficient (LogP)

Reversed‐phase TLC (RP‐TLC) plates from Macherey‐Nagel (Germany, Alugram Aluminiumfolien RP‐18W/UV_254_, stationary phase thickness: 0.15 mm) were used in this assay. Three mixtures of aqueous 3‐(N‐morpholino)propanesulfonic acid buffer (MOPS, 100 mM, pH 7.4) and ACN (3:2; 1:1; 2:3; v:v) were used as eluents. Naphthol (Log*P *= 2.85), benzophenone (Log*P *= 3.18), anthracene (Log*P *= 4.50) and perylene (Log*P *= 6.25) were used as known references [26]. Freshly prepared solutions of the samples and references (dissolved in acetone) were spotted on the plates. The solvent migration distance was 4.5 cm. The spots of compounds were monitored by using UV‐shadowing. Each spot was marked and its position relative to the start line was measured to determine *R*_f_ values. For boronic ester derivatives **7** and **21a,** two spots were observed corresponding to the initial ester and its hydrolyzed form. All measurements were done in triplicates. Obtained logP values: 5.39 ± 0.26 (**7**); 3.37 ± 0.24 (hydrolyzed form of **7**); 4.41 ± 0.32 (**21a**); 2.26 ± 0.10 (hydrolyzed form of **21a**); 3.83 ± 0.22 (**21b**); 3.07 ± 0.05 (**21c**).

### 4.6 Reaction of Compounds With H_2_O_2_ in Cell‐Free Settings

Degradation of the molecules upon oxidative treatment was assessed by HPLC coupled to a quadrupole (Q) mass detection (HPLC‐MS). Compounds were prepared as 3 mM stock solutions in DMSO and diluted with aqueous triethylammonium acetate buffer (TEAA, pH 7.4) containing 23% (v/v) ACN and 1% (v/v) DMSO to give a final compound concentration of 30 µM. Samples were divided into two equal portions: one portion was treated with H_2_O_2_ (10 mM), while an equal volume of H_2_O was added to the corresponding control samples. After incubation at 37 °C for 10 min, the samples were injected directly into the HPLC‐MS for analysis (Figure 6).

### 4.7 Monitoring Generation of Reactive Oxygen Species (ROS) in the Presence of Either Drugs or Control Compounds in Cell Free Settings

2′,7′‐Dichlorofluorescin diacetate (DCFH‐DA, 4.9 mg) was dissolved in *N*,*N*‐dimethylformamide (DMF, 100 µL) and mixed with aqueous NaOH solution (0.1 M, 900 µL). This mixture was incubated on a shaker for 30 min at 22 °C in the dark to obtain 2′,7′‐dichlorofluorescin (DCFH) stock solution (10 mM). Next, 3‐(*N*‐morpholino)propanesulfonic acid buffer (MOPS buffer, 100 mM, pH 7.4) containing *N*,*N*,*N’*,*N’*‐ethylenediaminetetraacetic acid (EDTA, 10 mM), glutathione (GSH, 5 mM), and H_2_O_2_ (10 mM) was prepared. Finally, DCFH was diluted in this buffer to 10 µM concentration. Monitoring of the fluorescence (*F*) was started (*λ*_ex_: 501 nm, *λ*_em_: 525 nm). After 5 min, the compound was added at the final concentration of 10 µM. Aqueous DMF (1%, v/v) was used as controls. The measurement was conducted for 300 min. The maximal initial increase of the fluorescence intensity ((dF/dt)_0_, min^−1^) was determined and used for comparison of the efficacies of drugs in the release of ROS in cell‐free settings.

### 4.8 Stability of Solutions of Compounds in DMSO

Compounds were dissolved in DMSO to a final concentration of 3 mM and stored either at 22 °C or at −20 °C. Stability was monitored over a period of 1 week. For each compound, an LC‐MS measurement was performed immediately after preparation of the DMSO stock solution (*t* = 0) to establish a reference profile. For LC‐MS analysis, aliquots of the DMSO stock solutions were diluted in ACN/water (15:85 v/v) prior to injection. All samples were stable at −20 °C, but decomposed when stored at 22 °C. Correspondingly, the compounds reported in this paper were stored at −20 °C.

### 4.9 Study of Uptake of Compounds

A2780 cells (6 × 10^6^ cells/µL) were seeded in T175 flasks (Greiner, Germany) in RPMI 1640 medium containing 10% FBS, 1% L‐glutamine, and 1% penicillin/streptomycin and incubated for 3 days at 37 °C in the chamber filled with 5% CO_2_. Next, the cells were washed with DPBS, and a fresh portion of RPMI 1640 medium (40 mL, 5% FBS, 1% L‐glutamine, 1% penicillin/streptomycin) was added. Compounds dissolved in DMSO were added into the bottles at final concentration of 10 µM (final DMSO concentration: 1%, v/v), and the cells were further incubated at either 37 °C or 4 °C for 4 h. Next, the cells were washed (2 × 10 mL DPBS) and trypsinated. After the cells were centrifuged (5 min, 1000 rpm), the cell pellet was mixed with concentrated HNO_3_ (100 µL) in an Eppendorf Thermomixer for 10 min at 95 °C. After cooling down, water (900 µL) was added, and the suspension was centrifuged. The supernatant was analyzed by atomic emission spectrometry (AES) using the Agilent 4200 MP‐AES (Agilent Technologies, Santa Clara, CA, USA): detection of B and Zn. Zn amount was used as an internal reference. Standard solutions for the AES were from Agilent or Bernd Kraft.

### 4.10 Determination of Intracellular Mitochondrial ROS (mROS)

Flow cytometry

A2780 cells were seeded in a 96‐well plate at a concentration of 250 cells/µL (total volume 100 µL per well) 1 day before the experiment in RPMI 1640 medium containing 5% FBS, 1% L‐glutamine, and 1% penicillin/streptomycin. The plate was left at 37 °C in the chamber filled with CO_2_ (5%) overnight. On the next day, the medium was replaced with the fresh one. Compounds, dissolved in DMSO (the final concentration: 25 µM, final DMSO amount: 1%, v/v), were added and incubated for 2 h. Next, the cells were washed (2 × 100 µL DPBS). MitoSOX (Thermo Fisher Scientific, stock solution in DMSO, 5 mM) was diluted (1:1000) in the Hank's Balanced Salt Solution (HBSS), added to the cells (total volume 100 µL), and incubated for 20 min in the incubator (5 µM final concentration). The cells were washed (2 × 100 µL DPBS), trypsinated, and resuspended in fresh medium (100 µL/well). The fluorescence intensity was measured using CytoFLEX, Beckmann Coulter: *λ*_ex_ = 488 nm, *λ*_em_ = 585 ± 42 nm; *N* = 3. The data were analyzed using CytExpert software.

Microscopy

A2780 and SBLF9 cells were seeded on a 35 mm imaging dish (*µ*‐Dish 35 mm, high, ibidi GmbH, Germany) at a cell density of 80 and 40 cells/µL, respectively, 1 day before the experiment in their corresponding growth medium (500 µL). Next day, the medium was replaced with a fresh one (2 mL, RPMI 1640 medium supplemented with 5% FBS, 1% L‐glutamine, and 1% penicillin/streptomycin) and compounds were added (2,5 mM stock solution, 25 µM final concentration in 2 mL) and incubated for 1 h. Then the cells were washed with PBS (2 × 2 mL) and MitoSOX (5 µM in DMSO) in HBSS (1 mL) were added and incubated for 20 min in the incubator. Finally, the cells were washed, and fresh medium (2 mL) was added. The images were taken with a Zeiss Axio Vert.A1 with objective: 40×/1.30 Oil DIC. A filter set for detection of MitoSOX: *λ*_ex_: 538–562 nm; *λ*_em_: 570–640 nm.

### 4.11 Mitochondrial Membrane Potential (MMP) Assay

A2780 cells were seeded in 96‐well plate at a concentration of 250 cells/µL with 100 µL per well 1 day before the experiment in RPMI 1640 medium containing 5% FBS, 1% L‐glutamine, and 1% penicillin/streptomycin, and the plate was left at 37 °C in the chamber filled with CO_2_ (5%) overnight. On the next day, the medium was replaced with fresh one. Compounds in DMSO were added at a final concentration of 25 µM and incubated for 2 h. Next, the cells were washed (2 × 100 µL DPBS). Rhodamine 123 (R123, Sigma Aldrich, stock solution 1 g/L in DMSO) was diluted (1:1000) in the Hank's Balanced Salt Solution (HBSS) and spread into the wells (100 µL) and incubated for 20 min in the incubator (100 µg/L final concentration). Next, the cells were washed (2 × 100 µL DPBS), trypsinated, and resuspended in fresh medium (100 µL/well). Fluorescence intensity was measured by using CytoFLEX, Beckmann Coulter: *λ*_ex_ = 488 nm, *λ*_em_ = 525 ± 40 nm; *N* = 3.

### 4.12 Determination of Intracellular Total ROS (tROS)

A2780 cells were seeded in a 96‐well plate at the concentration of 250 cells/µL (total volume 100 µL per well) 1 day before the experiment in RPMI 1640 medium containing 5% FBS, 1% L‐glutamine, and 1% penicillin/streptomycin. The plate was left at 37 °C in the chamber filled with CO_2_ (5%) overnight. On the next day, the cells were washed (2 × 100 µL DPBS). CM‐DCFH‐DA (Thermo Fischer, stock solution 2.5 mM in DMSO) was diluted (1:1000; final concentration 2.5 µM) in the Hank's Balanced Salt Solution (HBSS) and added to the cells (100 µL) and incubated for 20 min in the incubator. Next, the cells were washed (2 × 100 µL DPBS), and fresh medium was added (100 µL/well). Compounds, dissolved in DMSO (the final concentration: 25 µM, final DMSO amount: 1%, v/v), were added and incubated for 2 h. Finally, the cells were washed (2 × 100 µL DPBS), trypsinated, and the fluorescence intensity was measured using a CytoFLEX, Beckmann Coulter flow cytometer. Detection parameters: *λ*_ex_ = 488 nm, *λ*_em_ = 525 ± 40 nm; *N* = 3.

### 4.13 Determination of Long‐Term Oxidative Stress via DCCH Assay: Monitoring Intracellular Aldehydes

A2780 cells were seeded in 96‐well plate at a concentration of 250 cells/µL with 100 µL per well 1 day before the experiment in RPMI 1640 medium containing 5% FBS, 1% L‐glutamine, and 1% penicillin/streptomycin. The plate was left at 37 °C in the chamber filled with CO_2_ (5%) overnight. On the next day, the medium was replaced with fresh one. Compound **7** and **21a** in DMSO were added at final concentration of 1 and 5 µM (1% DMSO, v/v), and control NCure2—at 5 μM and incubated for 24 h. Next, the cells were washed (2 × 100 µL DPBS). DCCH (7‐diethylamino coumarin‐3‐carboxylic acid hydrazide, Sigma Aldrich, stock solution 80 mM in DMSO) was diluted (1:1000) in the Hank's Balanced Salt Solution (HBSS) and spread into the wells (100 µL) and incubated for 2 h in the incubator (80 µM final concentration). Next, the cells were washed (2 × 100 µL DPBS), trypsinated, and resuspended in fresh medium (100 µL/well). Fluorescence intensity was measured using CytoFLEX, Beckmann Coulter: *λ*_ex_ = 405 nm, *λ*_em_ = 450 ± 45 nm; *N* = 4. The data were analyzed using CytExpert software.

SBLF9 cells were grown and seeded in 24‐well plate (cell density: 40 cells/µL; 500 µL/well) in Ham's F‐12 Nutrient Mixture containing 15% FBS, 2% non‐essential amino acids, and 1% penicillin/streptomycin. The next day, the cells were washed twice, and a fresh portion of RPMI 1640 medium containing 5% FBS, 1% L‐glutamine, and 1% penicillin/streptomycin medium was added (500 µL). Compounds were added at final concentrations of 1 and 5 µM and incubated for 24 h. The rest of the experiment was conducted in the same way as described above for A2780 cells.

## Funding

This study was supported by Deutsche Forschungsgemeinschaft (MO 1418/18‐1), Horizon 2020 Framework Programme (FET Open: 861878, NeutroCure), Alexander von Humboldt‐Stiftung (Philipp Schwartz Initiative for a fellowship for RS).

## Conflicts of Interest

The authors declare no conflicts of interest.

## Supporting information

Characterization of new compounds is provided in the supporting information.

## Data Availability

The data that support the findings of this study are available on request from the corresponding author. The data are not publicly available due to privacy or ethical restrictions.
