# Supplementary material for: Hybrid Aminoferrocenes With Nitrate Esters for In Situ Peroxynitrite Generation: Complex Interplay Between Redox Chemistry, NO‐Donor Reactivity, and Aminoferrocene Activation Influences In Vitro Anticancer Activity
Source: Chembiochem. 2026 Jul 8;27(13):e70448. doi: 10.1002/cbic.70448 (PMC13343212; doi:10.1002/cbic.70448)
Supplement: Supplementary file 1 — Characterization of new compounds is provided in the supporting information. [file CBIC-27-e70448-s001.pdf]

SUPPORTING INFORMATION

Conjugates of Aminoferrocenes with a Nitrate ester: Synthesis and Anticancer activity in vitro

Hülya Gizem Özkan, Roman Selin, Paula Holst, Rainer Tietze, Christoph Alexiou and Andriy Mokhir

Content

	Page
Characterization of new compounds	S2

Characterization of the new compounds

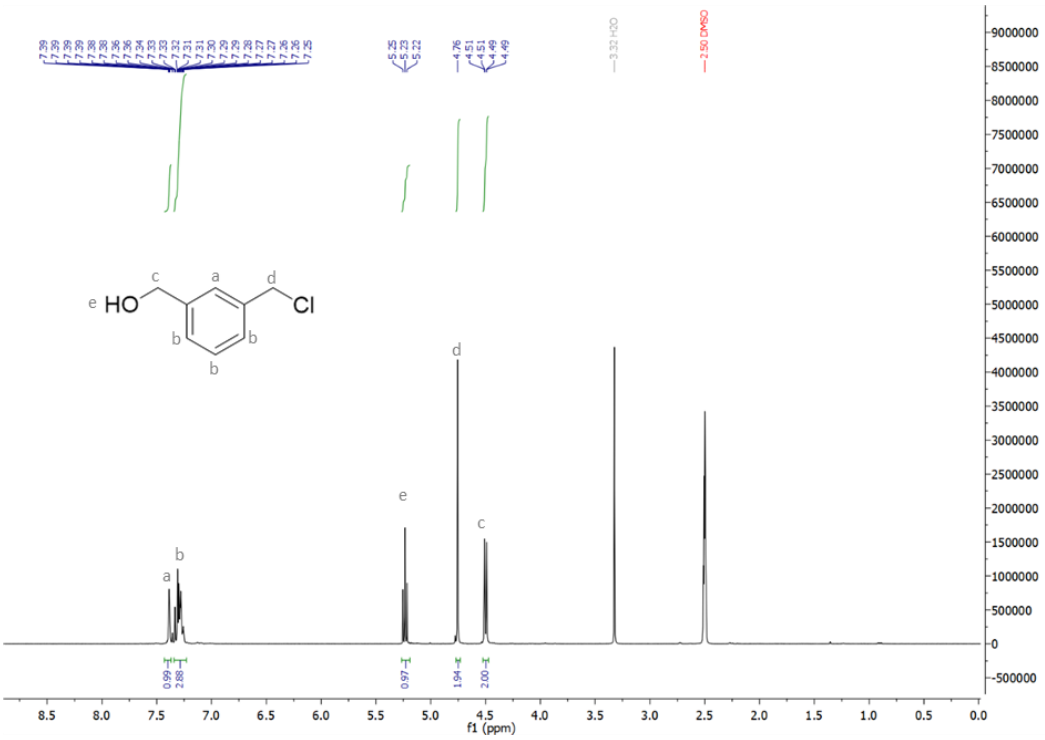

Figure S1. ^{1}H-NMR spectrum of **15** in DMSO-d_6.

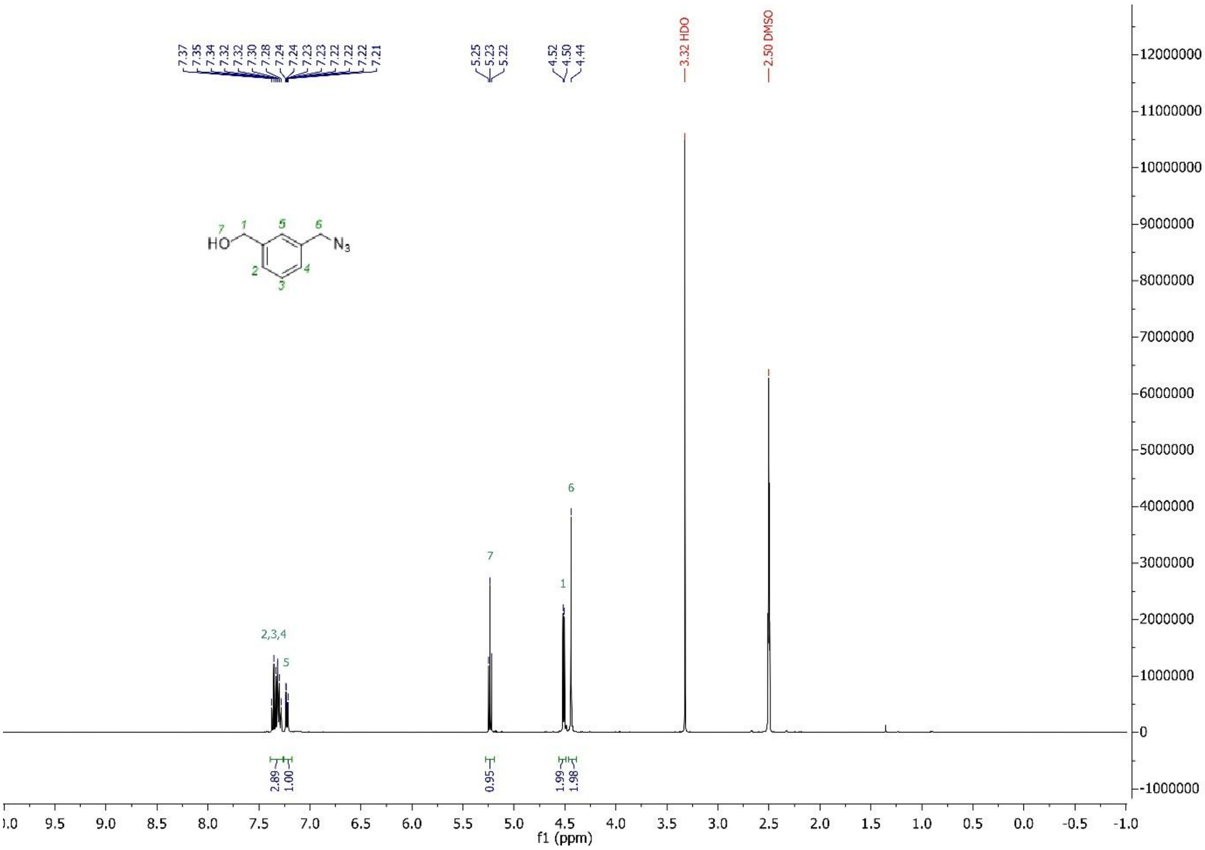

Figure S2. ^{1}H-NMR spectrum of **16** in DMSO-d_6.

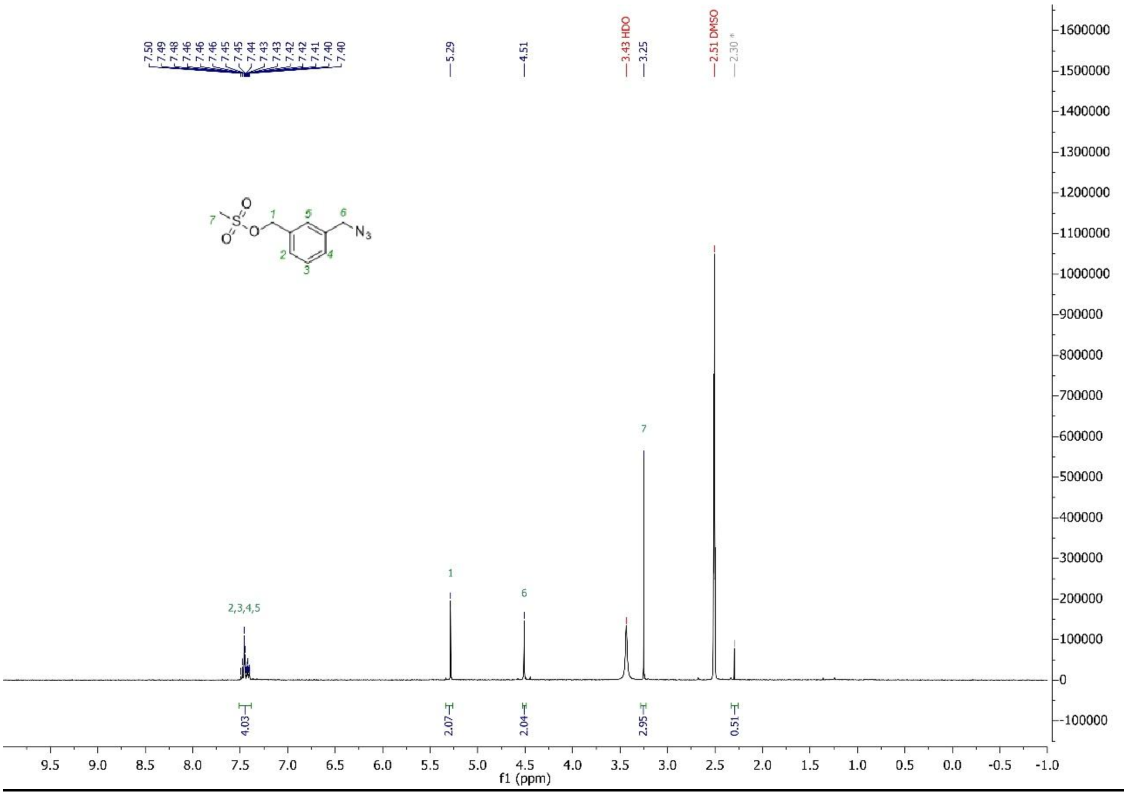

Figure S3. ^{1}H-NMR spectrum of **17** in DMSO-*d*$_6$.

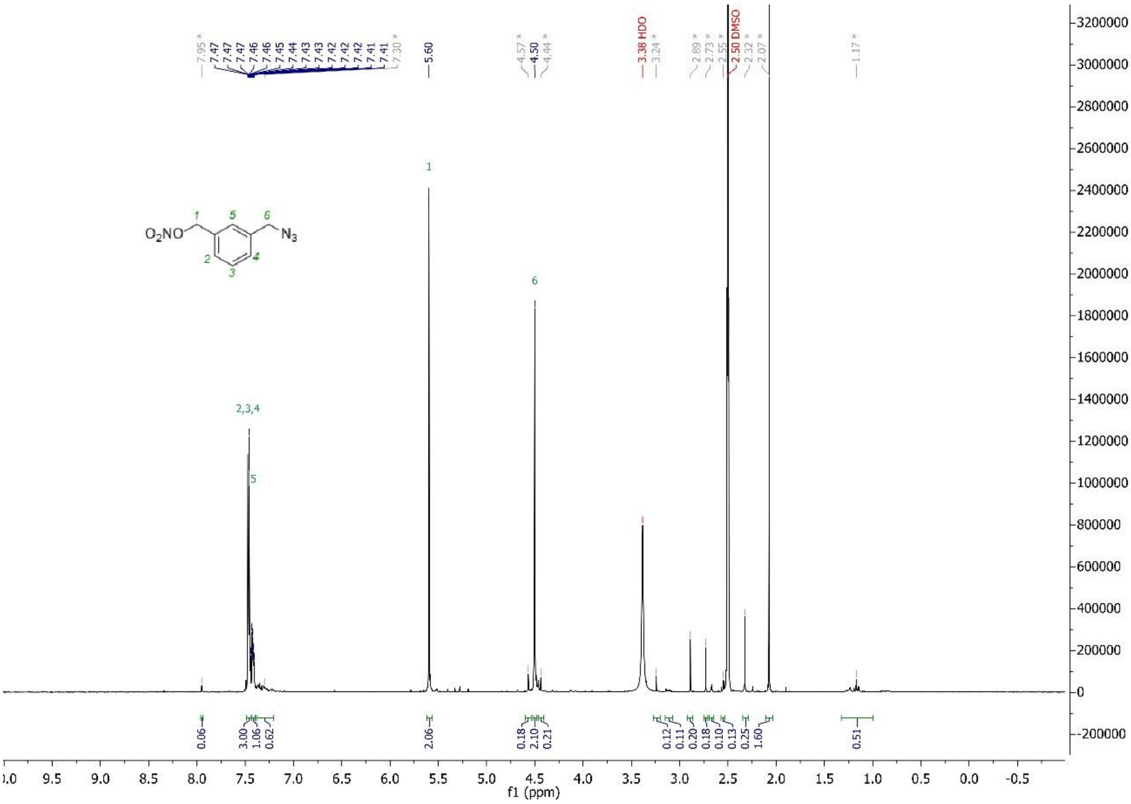

Figure S4. ^{1}H-NMR spectrum of intermediate **19** in DMSO-*d*$_6$.

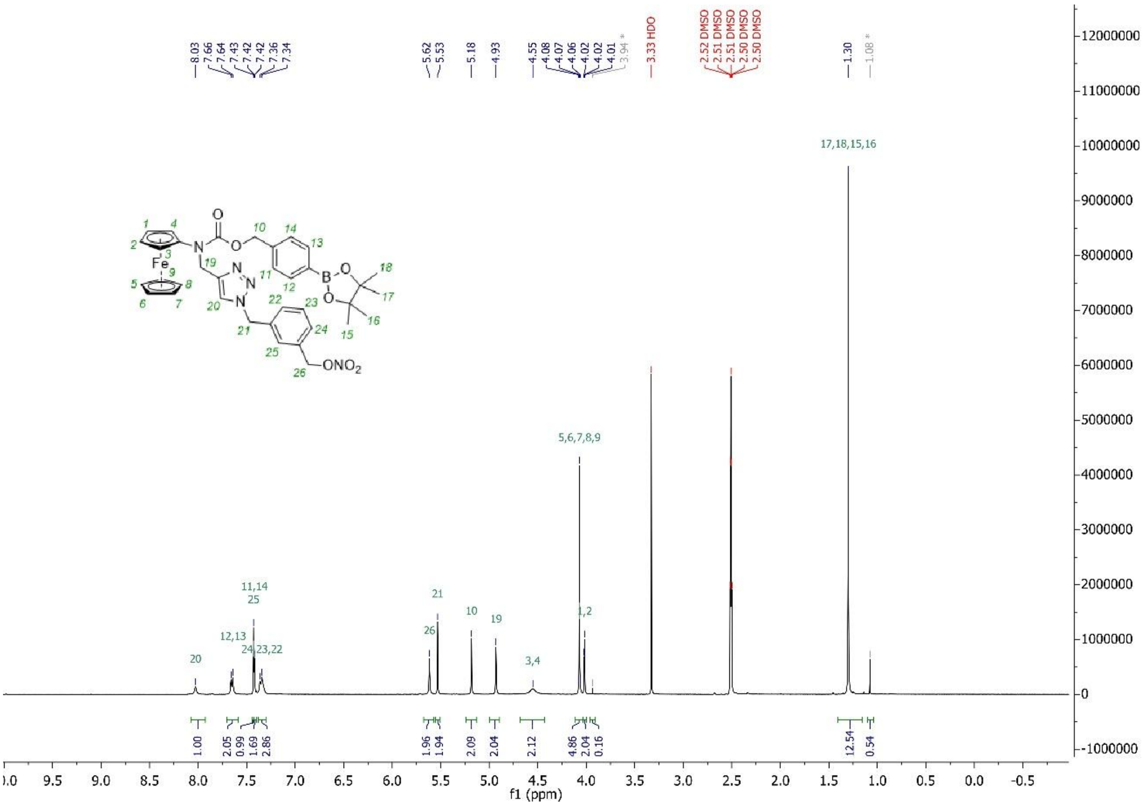

Figure S5. ^{1}H-NMR spectrum of prodrug **7** in DMSO-d_6.

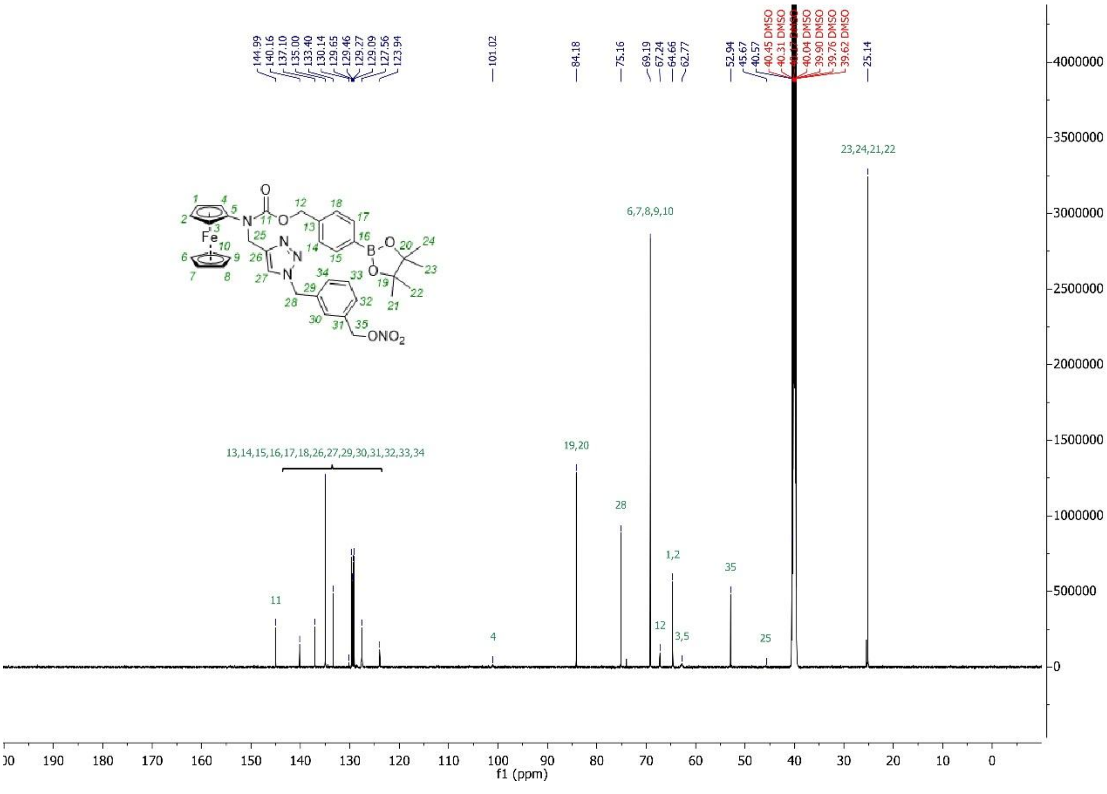

Figure S6. ^{13}C-NMR spectrum of prodrug **7** in DMSO-d_6.

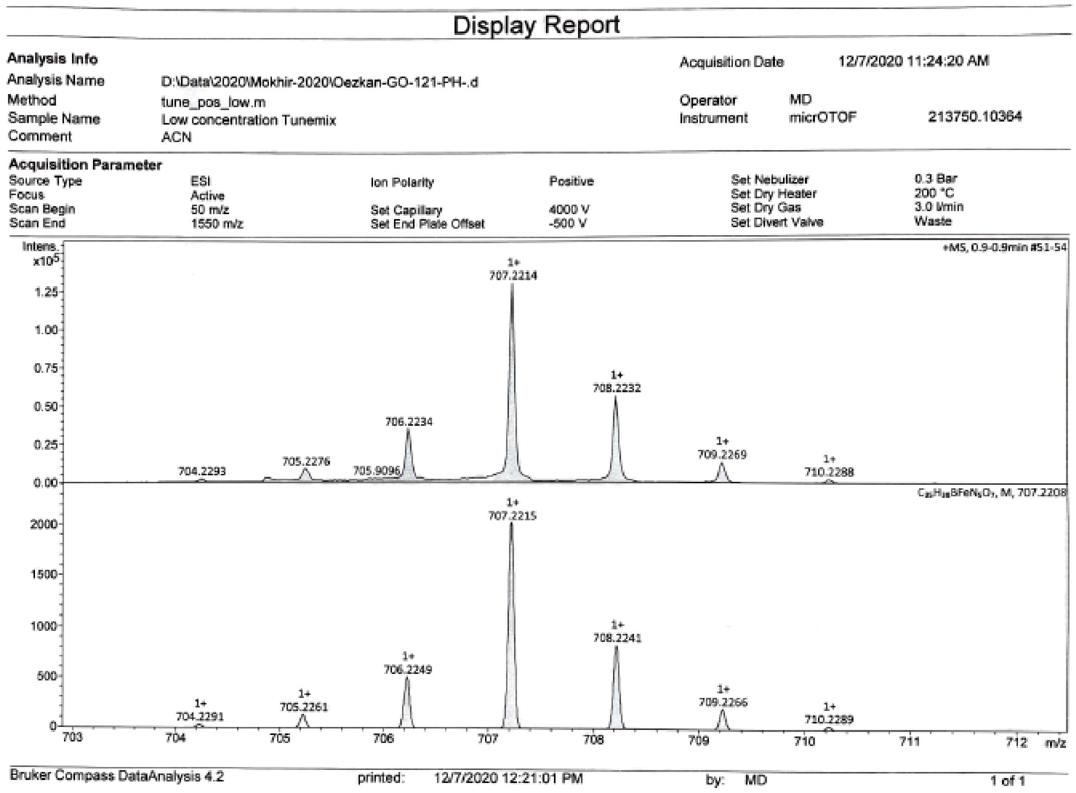

Figure S7. HR-MS spectrum of prodrug **7**: upper plot – experimental spectrum; bottom plot – theoretical spectrum.

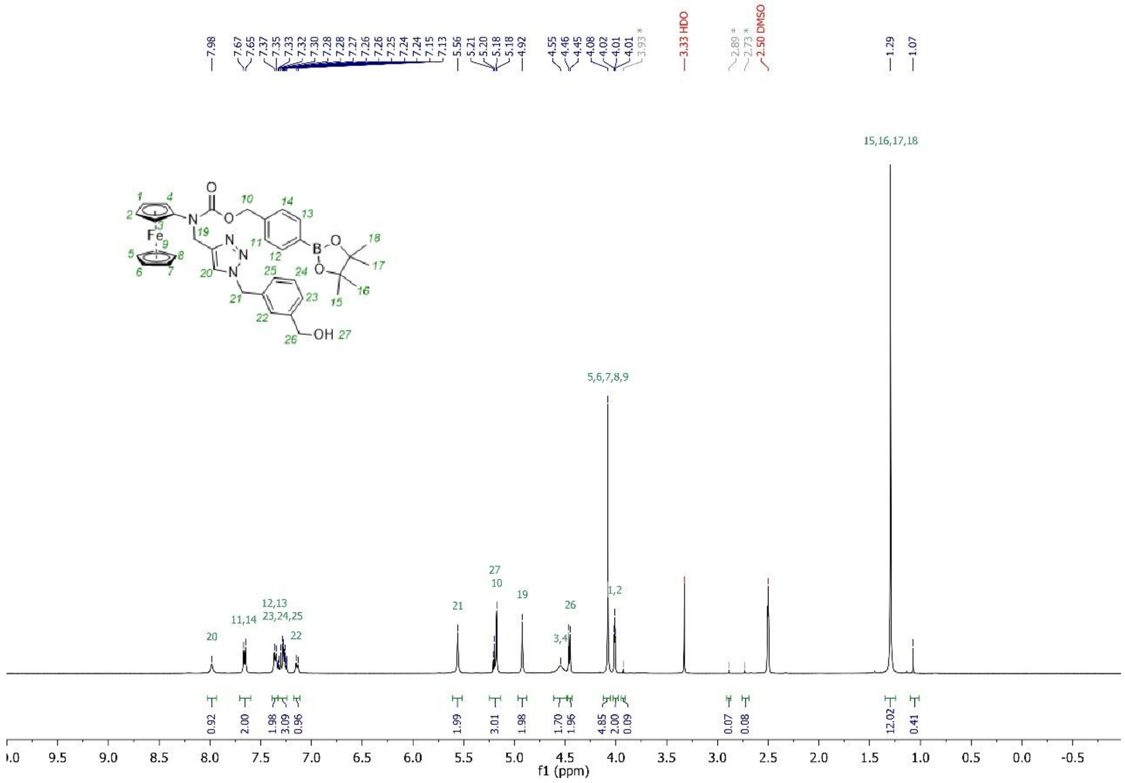

Figure S8. ^{1}H-NMR spectrum of **21a** in DMSO-d_6.

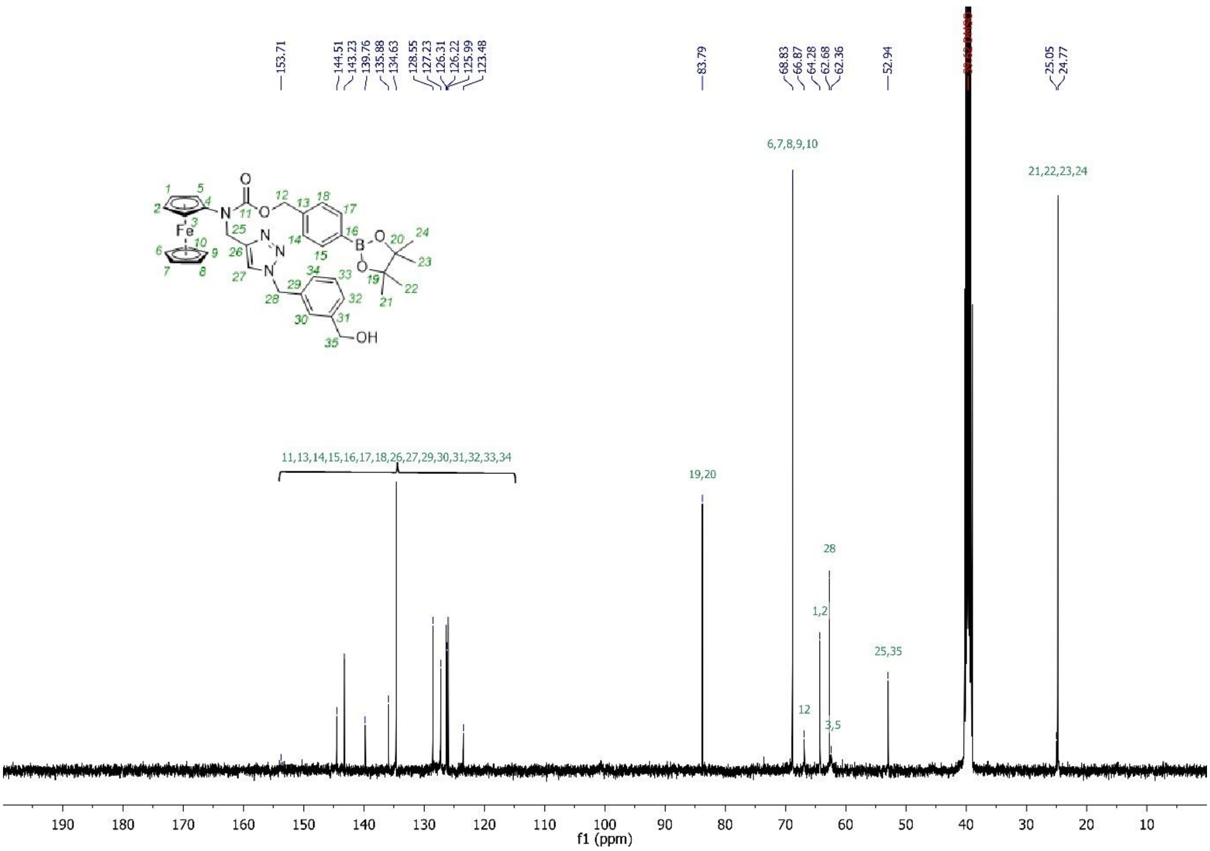

Figure S9. ^{13}C-NMR spectrum of **21a** in DMSO-d_6.

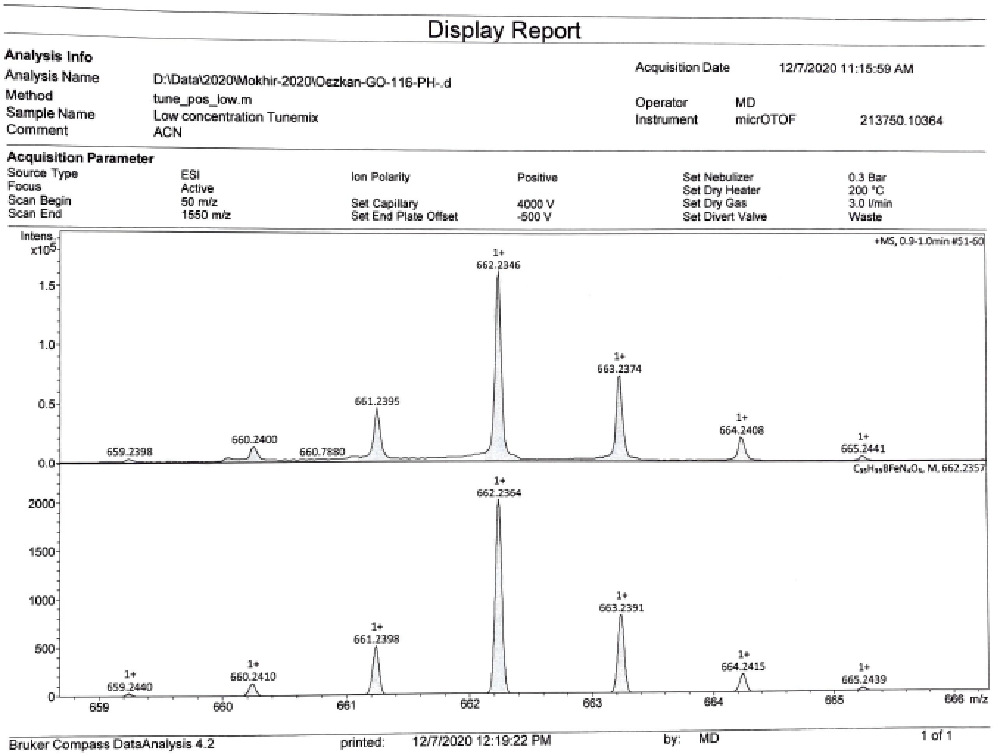

Figure S10. HR-MS spectrum of **21a**: upper plot – experimental spectrum; bottom plot – theoretical spectrum.

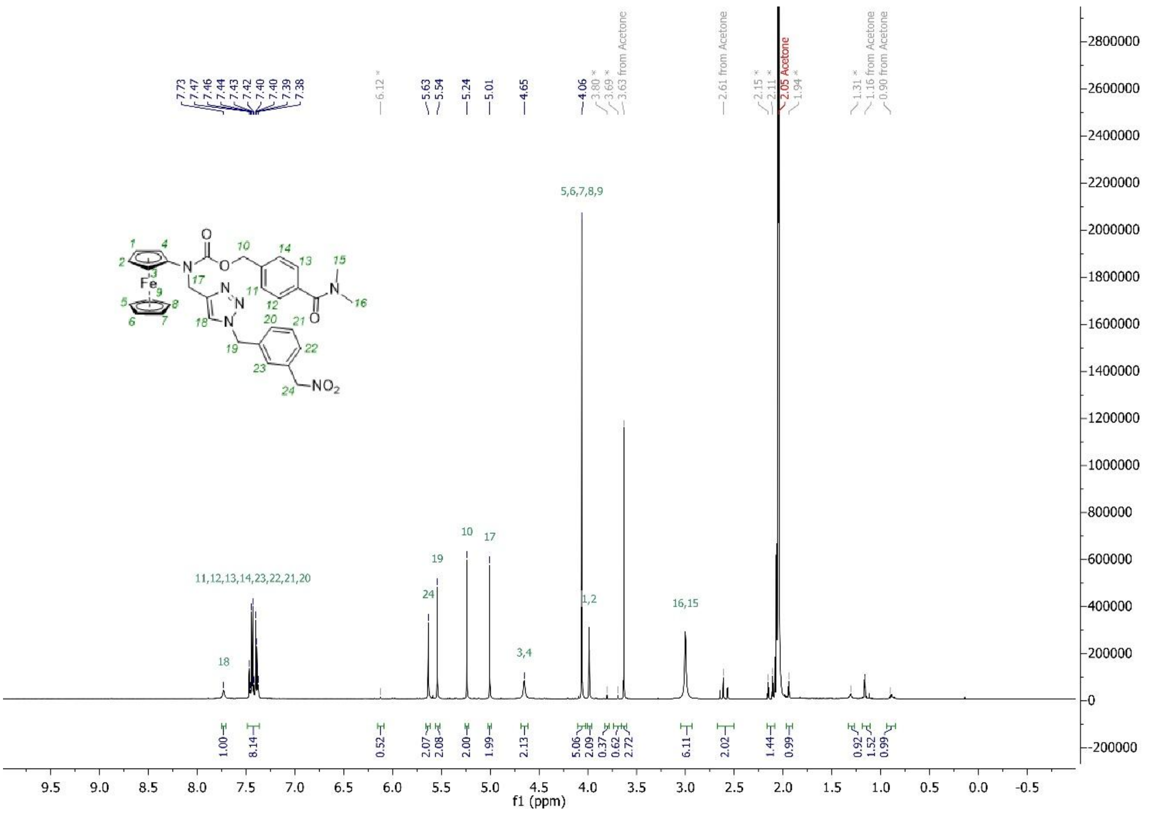

Figure S11. ^{1}H-NMR spectrum of **21b** in acetone-d_6.

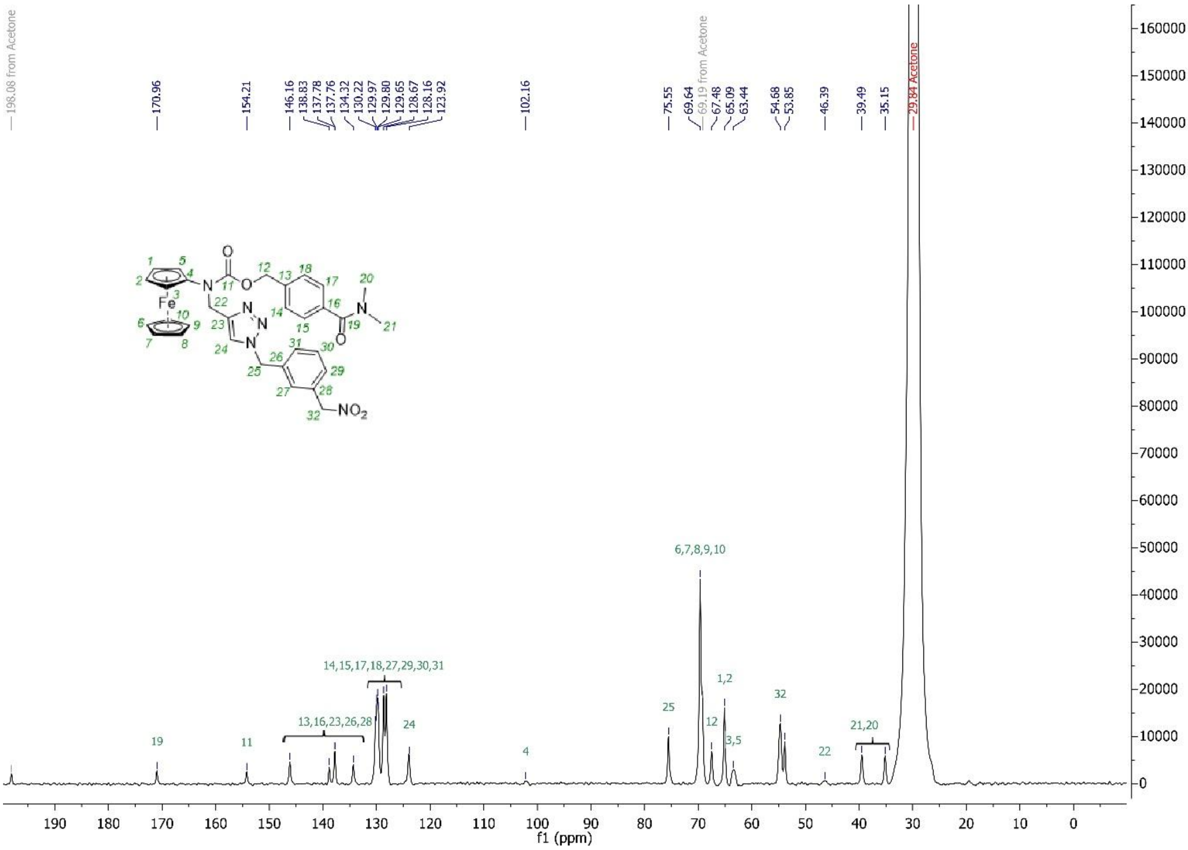

Figure S12. ^{13}C-NMR spectrum of prodrug **21b** in acetone-d_6.

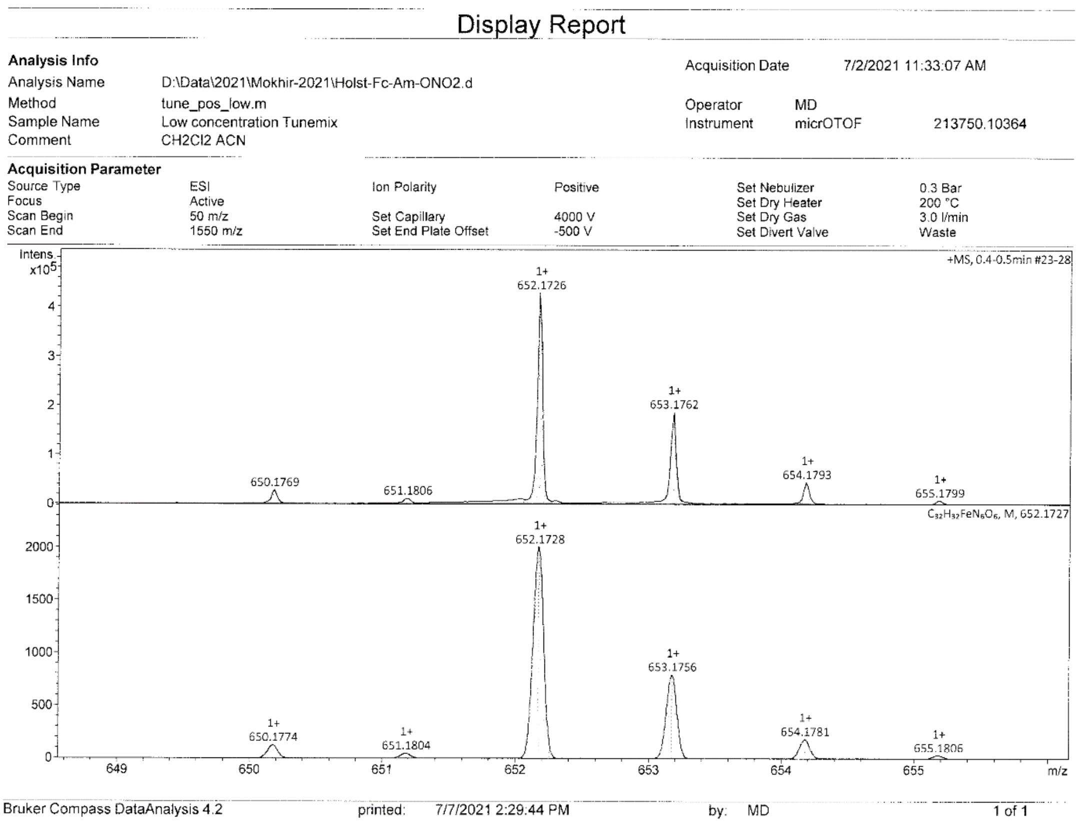

Figure S13. HR-MS spectrum of prodrug **21b**: upper plot – experimental spectrum; bottom plot – theoretical spectrum.

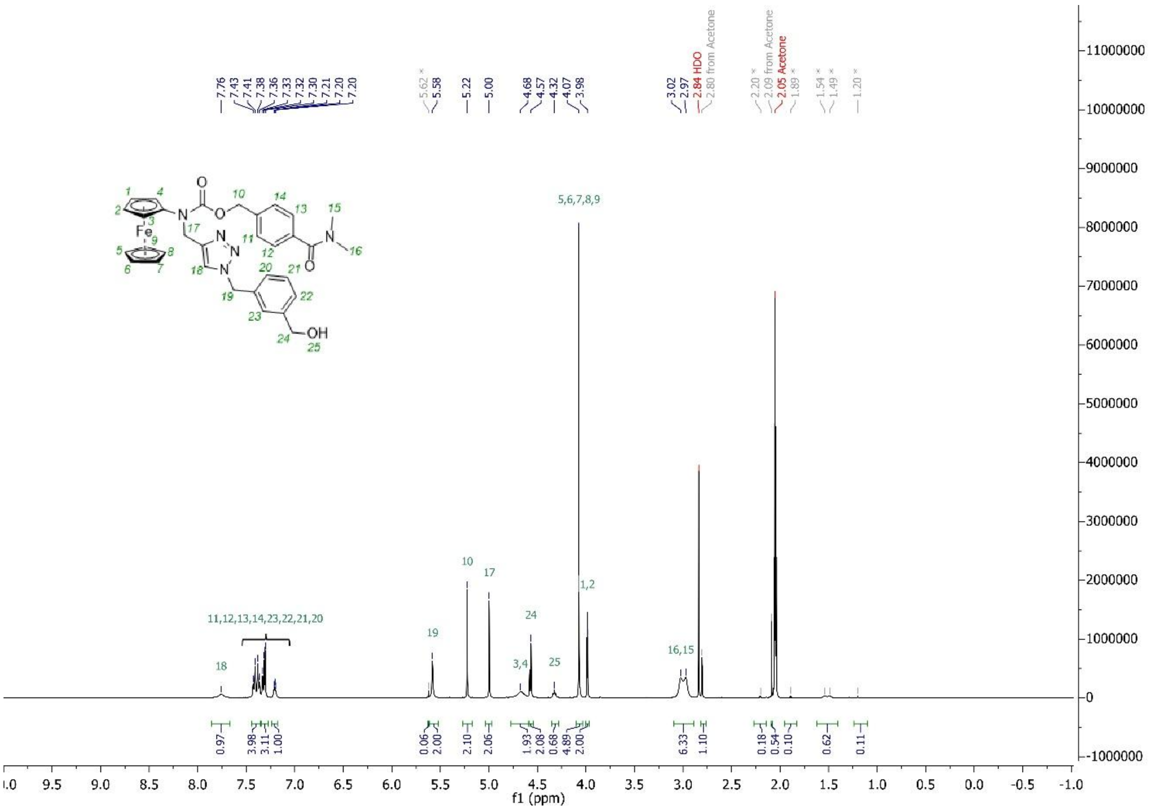

Figure S14. ^{1}H-NMR spectrum of **21c** in acetone-d_6.

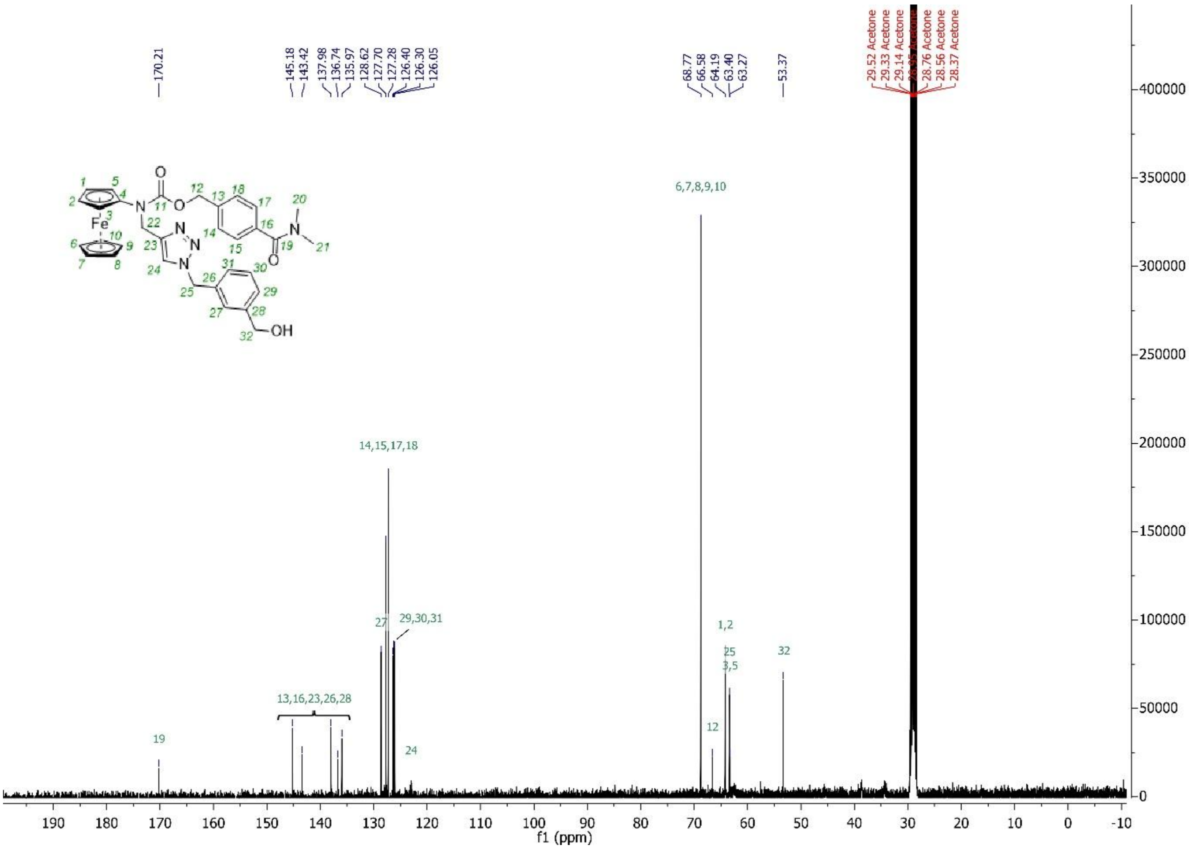

Figure S15. ^{13}C-NMR spectrum of prodrug **21c** in acetone-d_6.

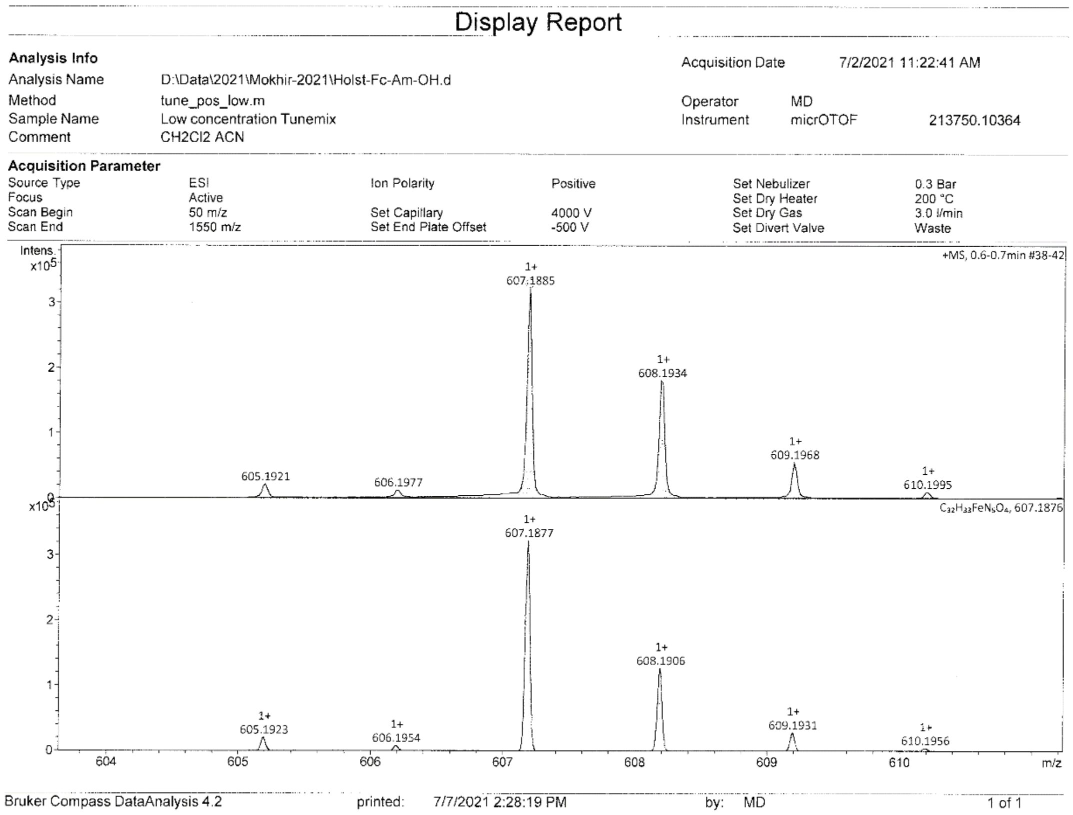

Figure S16. HR-MS spectrum of prodrug **21c**: upper plot – experimental spectrum; bottom plot – theoretical spectrum.
